# Linking sarcopenia, brain structure and cognitive performance: a large-scale UK Biobank study

**DOI:** 10.1093/braincomms/fcae083

**Published:** 2024-03-07

**Authors:** Tiril P Gurholt, Miguel Germán Borda, Nadine Parker, Vera Fominykh, Rikka Kjelkenes, Jennifer Linge, Dennis van der Meer, Ida E Sønderby, Gustavo Duque, Lars T Westlye, Dag Aarsland, Ole A Andreassen

**Affiliations:** Norwegian Centre for Mental Disorders Research (NORMENT), Division of Mental Health and Addiction, Oslo University Hospital and University of Oslo, Oslo 0424, Norway; Centre for Age-Related Medicine (SESAM), Stavanger University Hospital, Stavanger 4068, Norway; Faculty of Health Sciences, University of Stavanger, Stavanger 4036, Norway; Semillero de Neurociencias y Envejecimiento, Ageing Institute, Medical School, Pontificia Universidad Javeriana, Bogota 111611, Colombia; Norwegian Centre for Mental Disorders Research (NORMENT), Division of Mental Health and Addiction, Oslo University Hospital and University of Oslo, Oslo 0424, Norway; Norwegian Centre for Mental Disorders Research (NORMENT), Division of Mental Health and Addiction, Oslo University Hospital and University of Oslo, Oslo 0424, Norway; Norwegian Centre for Mental Disorders Research (NORMENT), Division of Mental Health and Addiction, Oslo University Hospital and University of Oslo, Oslo 0424, Norway; Department of Psychology, University of Oslo, Oslo 0373, Norway; AMRA Medical AB, Linköping 58222, Sweden; Department of Health, Medicine and Caring Sciences, Linköping University, Linköping 58183, Sweden; Norwegian Centre for Mental Disorders Research (NORMENT), Division of Mental Health and Addiction, Oslo University Hospital and University of Oslo, Oslo 0424, Norway; School of Mental Health and Neuroscience, Faculty of Health, Medicine and Life Sciences, Maastricht University, Maastricht 6200MD, The Netherlands; Norwegian Centre for Mental Disorders Research (NORMENT), Division of Mental Health and Addiction, Oslo University Hospital and University of Oslo, Oslo 0424, Norway; Department of Medical Genetics, Oslo University Hospital, Oslo 0424, Norway; Dr. Joseph Kaufmann Chair in Geriatric Medicine, Department of Medicine and Research Institute of the McGill University Health Centre, McGill University, Montreal, QC H4A 3J1, Canada; Norwegian Centre for Mental Disorders Research (NORMENT), Division of Mental Health and Addiction, Oslo University Hospital and University of Oslo, Oslo 0424, Norway; Department of Psychology, University of Oslo, Oslo 0373, Norway; Centre for Age-Related Medicine (SESAM), Stavanger University Hospital, Stavanger 4068, Norway; Department of Psychological Medicine, Institute of Psychiatry, Psychology, and Neuroscience, King's College London, London WC2R 2LS, UK; Norwegian Centre for Mental Disorders Research (NORMENT), Division of Mental Health and Addiction, Oslo University Hospital and University of Oslo, Oslo 0424, Norway

**Keywords:** degenerative conditions, ectopic fat, skeletal muscle, mediator, T_1_-weighted MRI

## Abstract

Sarcopenia refers to age-related loss of muscle mass and function and is related to impaired somatic and brain health, including cognitive decline and Alzheimer’s disease. However, the relationships between sarcopenia, brain structure and cognition are poorly understood. Here, we investigate the associations between sarcopenic traits, brain structure and cognitive performance. We included 33 709 UK Biobank participants (54.2% female; age range 44–82 years) with structural and diffusion magnetic resonance imaging, thigh muscle fat infiltration (*n* = 30 561) from whole-body magnetic resonance imaging (muscle quality indicator) and general cognitive performance as indicated by the first principal component of a principal component analysis across multiple cognitive tests (*n* = 22 530). Of these, 1703 participants qualified for probable sarcopenia based on low handgrip strength, and we assigned the remaining 32 006 participants to the non-sarcopenia group. We used multiple linear regression to test how sarcopenic traits (probable sarcopenia versus non-sarcopenia and percentage of thigh muscle fat infiltration) relate to cognitive performance and brain structure (cortical thickness and area, white matter fractional anisotropy and deep and lower brain volumes). Next, we used structural equation modelling to test whether brain structure mediated the association between sarcopenic and cognitive traits. We adjusted all statistical analyses for confounders. We show that sarcopenic traits (probable sarcopenia versus non-sarcopenia and muscle fat infiltration) are significantly associated with lower cognitive performance and various brain magnetic resonance imaging measures. In probable sarcopenia, for the included brain regions, we observed widespread significant lower white matter fractional anisotropy (77.1% of tracts), predominantly lower regional brain volumes (61.3% of volumes) and thinner cortical thickness (37.9% of parcellations), with |*r*| effect sizes in (0.02, 0.06) and *P*-values in (0.0002, 4.2e^−29^). In contrast, we observed significant associations between higher muscle fat infiltration and widespread thinner cortical thickness (76.5% of parcellations), lower white matter fractional anisotropy (62.5% of tracts) and predominantly lower brain volumes (35.5% of volumes), with |*r*| effect sizes in (0.02, 0.07) and *P*-values in (0.0002, 1.9e^−31^). The regions showing the most significant effect sizes across the cortex, white matter and volumes were of the sensorimotor system. Structural equation modelling analysis revealed that sensorimotor brain regions mediate the link between sarcopenic and cognitive traits [probable sarcopenia: *P*-values in (0.0001, 1.0e^−11^); muscle fat infiltration: *P*-values in (7.7e^−05^, 1.7e^−12^)]. Our findings show significant associations between sarcopenic traits, brain structure and cognitive performance in a middle-aged and older adult population. Mediation analyses suggest that regional brain structure mediates the association between sarcopenic and cognitive traits, with potential implications for dementia development and prevention.

## Teaser

Gurholt *et al*. report that traits relevant to sarcopenia, a muscle disorder linked to body and brain impairments, are associated with lower general cognitive performance and brain structure phenotypes. Sensorimotor brain structures mediated the link between sarcopenic and cognitive traits. The findings provide essential cues for understanding the sarcopenia–dementia link.

## Graphical Abstract

**Graphical Abstract:**
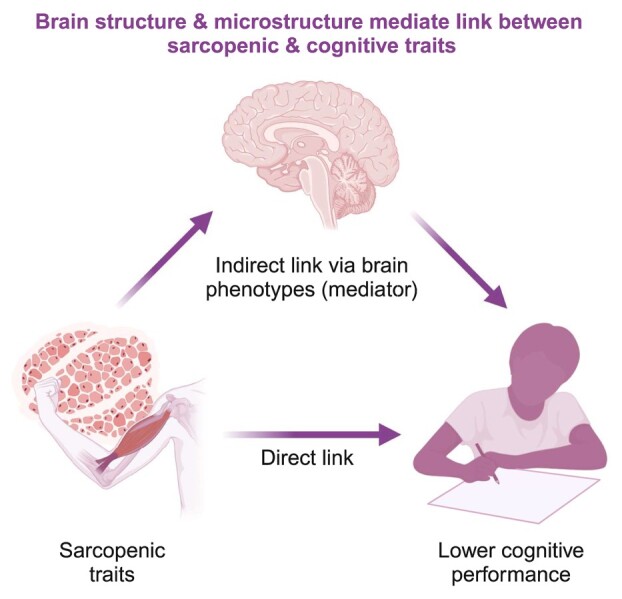

## Introduction

Sarcopenia is a geriatric condition of predominantly age-related loss of muscle mass and function,^1^ affecting 10–16% of the older population worldwide.^2^ Sarcopenia is associated with a wide range of adverse health outcomes,^2-4^ functional decline and reduced quality of life.^2^ Prior studies link sarcopenia or sarcopenic traits (e.g. lower physical performance and muscular strength, quantity and quality) with cognitive impairment^5-10^ and Alzheimer’s disease.^3,6,10^ Indeed, muscle function impairments may increase the risk for dementia.^11^ Despite this, the neurobiological underpinnings of the sarcopenia–dementia link are unclear, although increasing evidence suggests muscle–brain crosstalk involving mitochondrial, metabolic, inflammatory processes^5^ and myokines.^5,12^

While few prior studies exist, there are studies implicating parietal grey matter^13^ and white matter microstructural^14^ alterations amongst individuals with sarcopenia. Furthermore, sarcopenic traits, including lower muscular mass and strength, are associated with lower total or regional brain volumes.^6,13^ Higher muscle fat infiltration (MFI) from whole-body magnetic resonance imaging (MRI), which is an indicator of poor muscle quality, a component of sarcopenia,^1^ is associated with regionally thinner cortical thickness and lower cerebral and cerebellum grey matter volumes,^15^ muscular strength and function^16^ and all-cause mortality in adults.^17^ Additionally, ectopic muscle fat accumulation is associated with inflammation^18^ and oxidative stress,^19^ two processes related to muscle atrophy and sarcopenia^19^ and neurodegenerative conditions such as Alzheimer’s disease.^20^

Despite the observed associations between sarcopenia or sarcopenic traits, brain structure and cognitive impairments, to our knowledge, no prior studies have explicitly tested whether regional brain structure mediates the link between sarcopenia or sarcopenic traits and cognitive performance. Here, we use brain MRI (*n* = 33 709), thigh MFI (*n* = 30 561) from whole-body MRI and general cognitive performance (*n* = 22 530) measures of UK Biobank participants to investigate the relationships between two sarcopenic traits (probable sarcopenia versus non-sarcopenia and percentage of thigh MFI), brain structure and cognitive performance. We investigate whether and how probable sarcopenia and MFI separately relate to (i) general cognitive performance, (ii) brain grey and white matter structures and (iii) whether brain MRI phenotypes mediate the relationship between the two sarcopenic traits and cognitive performance.

## Materials and methods

### Participant sample

The UK Biobank is a large population cohort that captures the population from middle to old age and is well suited for studying conditions that arise in aging, such as sarcopenia, cognitive impairments and dementia. The UK Biobank performed the baseline recruitment of 500 000 participants during 2006–10, and the follow-up first imaging assessment began in 2014 and is ongoing. We included 33 709 UK Biobank participants (54.2% women) from the first imaging assessment with brain T_1_- and diffusion-weighted MRI data and relevant demographic and clinical data. Of these, 1703 participants (56.2% women) qualified for a probable sarcopenia diagnosis according to the *European Working Group on Sarcopenia in Older People* criteria (see below).^1^ A subset of participants had measures of general cognitive performance (*n* = 22 530) and percentage of MFI (*n* = 30 561) from whole-body MRI. We excluded participants who withdrew their informed consent (opt-out-list dated 22 February 2022).

The UK Biobank has ethics approvals from the Northwest Multi-Centre Research Ethics Committee and obtained informed consent from all participants.^21^ We have access to the UK Biobank resource through application number 27412.

### Demographic and clinical data

We extracted demographic (age, sex, ethnicity and education) and clinical data from the imaging time point from the UK Biobank repository. We included the relevant *sarcopenia-specific measures* [left/right handgrip strength (kg), bioimpedance left/right arm/leg fat-free mass (kg) and self-reported measures of inability to walk, walking speed and number of falls last year], *physical activity* (duration of walks, moderate and vigorous activity), *chronic neurological diagnosis*, *cardiometabolic risk factors* and available *cognitive test scores*. Prior studies^22-24^ describe the UK Biobank's fully automated self-administered touchscreen cognitive test battery. See Supplementary Notes 1–3 for the extracted Field-IDs, data cleaning, assessment and appendicular lean mass (ALM) estimation details.

We defined probable, confirmed and severe sarcopenia following the European Working Group on Sarcopenia in Older People criteria^1^ and prior UK Biobank studies^25,26^ as follows:

- *Probable sarcopenia*: low muscular strength, e.g. low handgrip strength, defined as <16 kg in women and <27 kg in men.^1^ Similar to prior studies,^25,26^ we used the maximum registered handgrip (left or right).
- *Confirmed sarcopenia*: probable sarcopenia AND low muscular quantity or quality.^1^ We evaluated muscular quantity based on low ALM/height^2^, defined as <5.5 kg/m^2^ in women and <7 kg/m^2^ in men.^1^
- *Severe sarcopenia*: confirmed sarcopenia AND low physical performance (e.g. low gait speed).^1^ Following prior studies,^25,26^ we approximated low gait speed by self-reported unable to walk or slow walking speed.
- *Non-sarcopenia:* remaining participants.

Due to low numbers of confirmed sarcopenia (*n* = 123), of which few qualified for severe sarcopenia (*n* = 11), we combined participants with probable, confirmed and severe sarcopenia into one *probable sarcopenia group* characterized by low handgrip strength—in line with prior studies^25,26^—for the statistical analyses.

We summarize the demographic and clinical data for the sample split on sex (Supplementary Table 1) and diagnosis (probable sarcopenia versus non-sarcopenia group; Supplementary Table 2).

### Brain and whole-body MRI acquisition

Previous studies have described the UK Biobank MRI acquisition protocol in detail.^21,27-29^ Briefly, UK Biobank acquired brain T_1_- and diffusion-weighted MRI data using a 3T Siemens Skyra scanner and whole-body MRI data using a 1.5T Siemens MAGNETOM Aera scanner. We included MRI data from three assessment sites (Cheadle, Reading and Newcastle). The assessment of individual participants takes place on the same day and site.

### Brain and whole-body MRI post-processing

We obtained and processed raw brain DICOM images using standardized protocols outlined below, yielding total control of the processing pipeline and data quality.

We processed T_1_-weighted brain MRI data using FreeSurfer^30^ (version 5.3.0; http://www.freesurfer.net). We extract the regional cortical thickness and area from all 34 parcellations of the Desikan–Killiany cortical atlas^31^ (Supplementary Fig. 1) and 20 brain volumes from the aseg atlas, namely, cerebellum grey and white matter, brainstem, thalamus, hippocampus, amygdala, accumbens, caudate, putamen, pallidum, ventral diencephalon, corpus callosum (anterior, central, mid anterior, mid posterior and posterior), cerebrospinal fluid and lateral, third, and fourth ventricular volumes (Supplementary Fig. 2). For bilateral structures, we extracted measures of the left and right hemispheres. Additionally, we extracted Euler numbers from the cortical reconstruction^32^ as a proxy for image quality.^33^

We processed the diffusion-weighted MRI data using an optimized post-processing pipeline^34^ (Supplementary Note 4) and derived the diffusion maps for fractional anisotropy (FA). We applied *tract-based spatial statistics* to extract diffusion metrics,^35^ created the mean FA skeleton and projected the individual FA maps onto the mean FA skeleton. We extracted 27 regions of interest (Supplementary Table 3) across both hemispheres from the Johns Hopkins University labelled DTI atlas.^36^

As provided by the UK Biobank, we included thigh MFI derived from the whole-body MRI data processed by AMRA (AMRA Medical AB, Linköping, Sweden; https://www.amramedical.com). Briefly, AMRA derives MFI measures as T_2_*-corrected fat fraction of the ‘fat-free muscle volume’ (i.e. the total volume of image voxels with fat fraction < 50%^37^) of the anterior and posterior thighs.^38,39^ The analysis includes image calibration using fat-referenced MRI, automatic atlas-based segmentation, manual quality control and quantification of fat/muscle volumes and fat fractions. We extracted measures of anterior and posterior thigh MFI [percentage (%)] and computed their mean, denoted as MFI.

### Quality control

**Figure 1:**
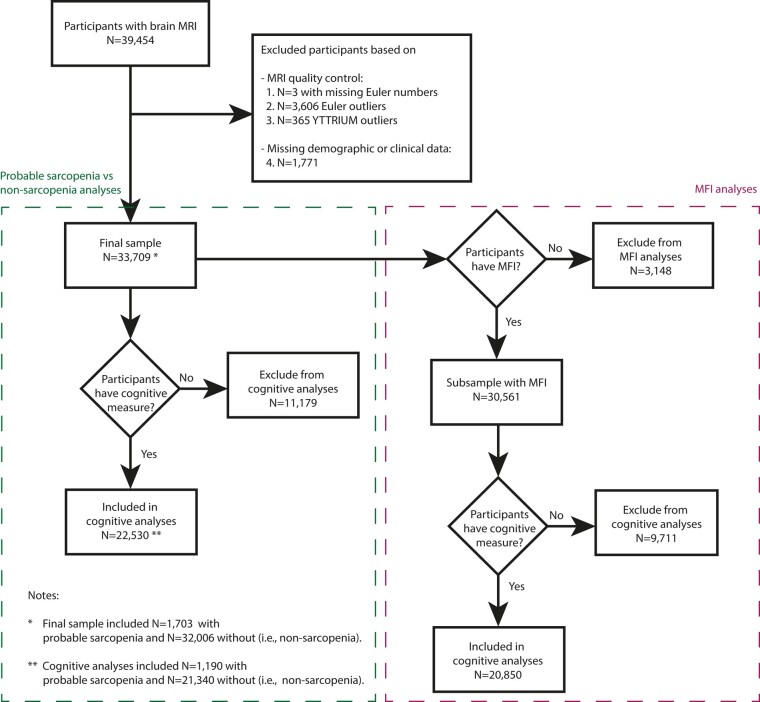
**Flowchart of participant inclusion process.** We performed subsample analyses for the participants with a measure of total thigh MFI in percentage and for those with a measure of general cognitive performance (PC1, first principal component). YTTRIUM, Fast qualitY conTrol meThod foR derIved diffUsion Metrics.

Initially, we had 39 454 participants with T_1_- and diffusion-weighted brain MRI data. Due to the large number of participants, we used automated MRI data quality control. For T_1_-weighted MRI, we first excluded three participants with missing Euler numbers before iteratively removing^15^ 3606 participants labelled as Euler outliers.^33^ We iteratively defined outliers as higher negative Euler numbers exceeding three standard deviations from the mean in either hemisphere until no outliers remained (six iterations).^15^ Subsequently, we excluded 365 participants detected as diffusion MRI outliers based on YTTRIUM.^40^ In total, we removed 3971 participants due to poor image quality on either T_1_- or diffusion-weighted MRI. Amongst the remaining 35 480 participants, we removed 1771 due to incomplete demographic or clinical data, yielding the final sample of 33 709 participants. The subsample with a measure of thigh MFI in percentage from whole-body MRI consisted of 30 561 participants (Fig. 1).

### General cognitive performance

The UK Biobank cognitive test battery consists of several correlated cognitive tests (Supplementary Fig. 3A) that cover partly overlapping aspects of cognition. We did not have an a priori hypothesis regarding which specific cognitive test was more relevant for sarcopenic traits. Thus, we derived a data-driven measure of general cognitive performance using principal component analyses.^41^ All cognitive tests contributed to the first principal component (PC1), which we use to assess general cognitive performance. Principal component analysis is sensitive to outliers.^42^ Therefore, we iteratively removed the cognitive test statistics that exceeded three standard deviations from the mean until no outliers remained. Per cognitive test, we removed 32–1116 participants using 1–9 iterations.

Post outlier removal, we had *n* = 31 961 participants with cognitive data, of which *n* = 13 544 participants (42.4%) had missing data points. Therefore, we imputed the missing data using the iterative *regularized principal component analysis method* (missMDA R-package) until convergence, taking similarities between individuals and links between variables into account while avoiding overfitting.^43,44^ We included participants with at least 50% valid cognitive tests (i.e. ≥5 tests of 9) to maximize the preserved variance. For imputation, we included *n* = 22 530 participants, with a *2.5%* total variable missingness.

After imputation, we performed the principal component analysis on centred and scaled cognitive data (*prcomp* R-function). We extracted PC1 as an indicator of general cognitive performance.^41^ PC1 explained 36.9% of the total variability of the cognitive data across the nine cognitive tests (Supplementary Fig. 3B). We observed the largest PC1 loadings for symbol digits substitution (−0.37) and alphanumeric trail making (0.43), but all cognitive tests contributed to PC1 (Supplementary Fig. 3C). The biplot indicates negative correlations between PC1 and test statistics that count the number of correct answers (contributes negatively to PC1) and positive correlations with the remaining tests where a higher score indicates poorer performance (contributes positively to PC1). Overall, a higher score on PC1 indicates poorer general cognitive performance. To facilitate interpretability, we inverted PC1 so that a lower PC1 corresponds to lower general cognitive performance in the subsequent analyses.

Finally, the participant subsample with a measure of general cognitive performance (PC1) consisted of *n* = 22 530 participants, of which *n* = 1190 were in the probable sarcopenia group, and the remaining *n* = 21 340 were in the non-sarcopenia group (Fig. 1). The subsample with measures of MFI (%) and PC1 consisted of *n* = 20 850 participants (Fig. 1).

### Statistical analysis

We evaluated sample demographics across and within sexes (Supplementary Table 1) and probable sarcopenia and non-sarcopenia (Supplementary Table 2) and the normality of all continuous variables (split on sex; data not shown). We assessed density plots for expected distribution patterns of included brain structures (data not shown).

In the *primary analyses*, we used multiple linear regression to investigate how sarcopenic traits (probable sarcopenia relative to non-sarcopenia and MFI in percentage) relate to (i) general cognitive performance (PC1) and (ii) regional brain structure and microstructure (i.e. cortical thickness/area, brain volumes and white matter FA). We performed sensitivity analyses to verify that the results from the probable sarcopenia versus non-sarcopenia analyses were not driven by the participant subset with confirmed sarcopenia. We log-transformed ventricular volumes since the residuals violate the normality criteria of linear regression.^15^ We included sex, age, age^2^, sex-by-age, sex-by-age^2^, body mass index, European ancestry (yes/no), self-reported metabolic/lifestyle variables [cigarette smoker (yes/no), alcohol consumer (yes/no), diabetes (yes/no), hypertension (yes/no) and hypercholesterolaemia (yes/no)], higher education (yes/no) and assessment site in all linear models. For T_1_-weighted MRI, we additionally adjusted for the intracranial volume (except cortical thickness) and average Euler number (image quality proxy). We entered categorical variables as factors and mean-centred continuous variables.

We adjusted for sex, age, age^2^, sex-by-age and sex-by-age^2^ because it is known that there are sex- and age-related differences in brain structure.^45,46^ We adjusted for ethnic ancestry because sarcopenic traits may be sensitive to ethnic origin or related socioeconomic factors,^47^ higher education because it may be a protective factor for the development of cognitive impairments,^48^ body mass index because it is correlated with muscle mass and is a general estimate of body shape and size^1^ and other lifestyle/metabolic factors because they are associated with sarcopenic traits^49^ or brain structure phenotypes.^15^ Lastly, when applicable, we adjusted for relevant and common neuroimaging covariates (i.e. assessment site, intracranial volume and image quality).

We included *mediation analyses*^50^ using the Lavaan structural equation modelling (SEM) framework^51,52^ to investigate whether brain structure mediates the link between sarcopenic traits (probable sarcopenia versus non-sarcopenia and MFI in percentage) and general cognitive performance (PC1) using the following equations:


$$M_{\mathrm{brain}}=i_{M}+aX_{\mathrm{sarcopenic}\;\mathrm{trait}}+\mathrm{covariates}+e_{M},$$

$$Y_{\mathrm{cognition}}=i_{Y}+c^{\prime }X_{\mathrm{sarcopenic}\;\mathrm{trait}}+bM_{\mathrm{brain}}+\mathrm{covariates}+e_{Y}.$$

The model includes the outcome (*Y*_cognition_), mediator (*M*_brain_), intercepts (*i_M_*, *i_Y_*), inputs (*X*_sarcopenic trait_), covariates and error terms (*e_M_*, *e_Y_*). To limit the number of tests while still validating whether brain structure mediates the link between sarcopenic and cognitive traits, we restricted the analyses to the subset of brain regions with the most significant associations in the primary analyses of probable sarcopenia relative to non-sarcopenia—within the cortical thickness, brain volumes and white matter FA analyses—that were also significant in the corresponding primary analyses of MFI. Additionally, we included the lateral ventricle, which was amongst the most significant in the primary MFI analyses and reaches significance in the corresponding analyses of probable sarcopenia relative to non-sarcopenia. The mediation analyses provide us with estimates of a direct, an indirect (via brain structure) and a total effect of sarcopenic traits on general cognitive performance (PC1). We included similar covariates as above but coded *exogenous categorical variables* (i.e. independent categorical variables) as numeric dummy 0/1 variables and omitted interaction terms. The assessment site variable, consisting of three sites, was coded using two dummy variables.^53^ We implemented the mediation analyses with bootstrap standard errors, computed using standard bootstrapping^51^ and 10 000 bootstrap draws.

We implemented all statistical analyses in R (version 4.1.2; https://www.r-project.org). We used *lm* for the multiple linear regression analyses. We computed the partial correlation coefficient’s *r* effect size directly from the *t*-statistics for continuous variables and via Cohen’s *d* for categorical variables.^54^ We used SEM (Lavaan R-package;^51,52^ version 0.6-9) for the mediation analyses and reports estimates, *z*-scores and *P*-values. Given the exploratory nature of this study, we stringently adjusted for multiple comparisons using Bonferroni correction at *α* = 0.05 across *n* = 232 independent tests yielding a *study-wide significance threshold* of *P* ≤ *α/N* = 0.0002 (rounded to four decimal points). *N* = (*n*_1_ + *n*_2_ + *n*_3_ + 1) × *n*_4_ is the number of included cortical parcellations (*n*_1_ = 34 × 2; thickness and area), deep and lower brain volumes (*n*_2_ = 20), white matter microstructure regions of interest (*n*_3_ = 27) and sarcopenic traits (*n*_4_ = 2), counting bilateral regions and partly overlapping models once. We here focus on the most significant findings but offer the full results in the supplemental material (Supplementary Tables 4–17).

## Results

### Demographics

The sample included 33 709 participants [18 285 (54.2%) women, mean age 62.7 ± 7.3 years; 15 424 (45.8%) men, mean age 63.7 ± 7.5 years]. On average, women had lower handgrip strength, higher MFI and overall lower cardiometabolic risk than men (Supplementary Table 1). A total of 1703 participants (5.1% of the sample; 56.2% women) qualified for probable sarcopenia, which included *123* participants with confirmed sarcopenia (22.8% women), of which 11 qualified for severe (18.2% women) sarcopenia. The *probable sarcopenia* group (*n* = 1703) had, as expected, on average, higher MFI and lower ALM relative to *non-sarcopenia* (*n* = 32 006). Additionally, on average, the probable sarcopenia group had higher age and prevalence of self-reported diabetes, hypercholesterolaemia and hypertension but lower height and weight and fewer alcohol consumers. Self-reported chronic neurological conditions were slightly more prevalent in the probable sarcopenia group (1%) than in the non-sarcopenia group (0.4%) (Supplementary Table 2).

### General cognitive performance

Our analyses revealed that probable sarcopenia (*n* = 1190) was associated with lower general cognitive performance (PC1) (*r* = −0.04, *P* = 7.3e^−10^) relative to non-sarcopenia (*n* = 21 340). Similarly, in the participants, subsample with MFI and PC1 (*n* = 20 850) measures, higher MFI was also associated with lower general cognitive performance *(r* = −0.05, *P* = 2.1e^−13^).

### Brain structure in probable sarcopenia

**Figure 2:**
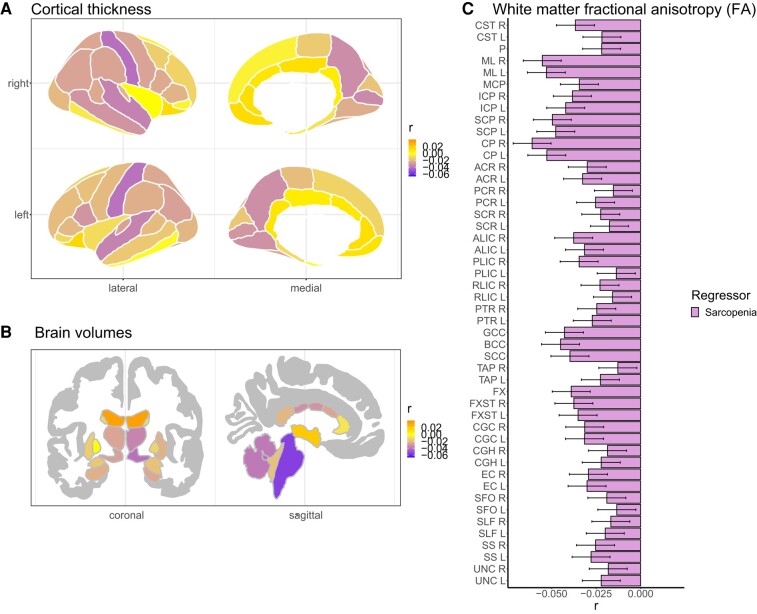
**Brain structure differences between individuals with probable sarcopenia versus non-sarcopenia.** We used multiple linear regression to obtain the partial correlation *r* effect size maps for probable sarcopenia (*n* = 1704) relative to non-sarcopenia (*n* = 32 171) for (**A**) cortical thickness, (**B**) brain volumes and (**C**) white matter FA. For white matter FA, we additionally display the 95% confidence interval. Significant *r* effect sizes are |*r*| in (0.02, 0.06) and *P*-values in (0.0002, 4.2e^−29^). We adjusted for sex, age, age^2^, sex-by-age, sex-by-age^2^, body mass index, ancestry, metabolic/lifestyle variables, higher education, site, ICV (except FA and cortical thickness) and Euler numbers (T_1_-weighted MRI). L, left; R, right; *r*, partial correlation coefficient; *Brainstem tracts*: CST, corticospinal tract; ML, medial lemniscus; P, pontine; MCP, middle cerebellar peduncle; ICP, inferior cerebellar peduncle; SCP, superior cerebellar peduncle. *Projection pathways*: CP, cerebral peduncle; ACR, anterior corona radiata; PCR, posterior corona radiata; SCR, superior corona radiata; ALIC, anterior limb of the internal capsule; PLIC, posterior limb of the internal capsule; RLIC, retrolenticular part of the internal capsule; PTR, posterior thalamic radiation. *Commissural pathways*: BCC, body of corpus callosum; GCC, genu of corpus callosum; SCC, splenium of the corpus callosum; TAP, tapetum. *Association pathways:* FX, fornix; FXST, fornix stria terminalis; CGC, cingulum cingulate gyrus; CGH, cingulum (hippocampal portion); EC, external capsule; SFO, superior fronto-occipital fasciculus; SLF, superior longitudinal fasciculus; SS, sagittal stratum; UNC, uncinate fasciculus.

Overall, in probable sarcopenia (*n* = 1704) relative to non-sarcopenia (*n* = 32 171), we found *86* study-wide significant brain structural and microstructural phenotypes across both hemispheres (Fig. 2; Supplementary Tables 4–7), amounting to 40% of the investigated regions across cortical thickness and area, brain volumes and microstructural white matter FA. The observed significant differences were small, with significant *r* effect sizes |*r*| in (0.02, 0.06) and *P*-values in (0.0002, 4.2e^−29^).

The analyses revealed regionally thinner cortex in individuals with probable sarcopenia relative to non-sarcopenia for 27 (i.e. 37.9%) of 68 cortical parcellations across both hemispheres (Fig. 2A; Supplementary Table 4), with the largest effect sizes in the post-central (left/right *r* = −0.04, *P* = 2.2e^−13^/7.0e^−14^) and superior temporal (left/right *r* = −0.04, *P* = 7.8e^−11^/5.8e^−11^) gyri of the parietal and temporal lobes. There was limited evidence of cortical area differences, with only three regions—the left fusiform (*r* = −0.02, *P* = 9.2e^−05^), left inferior parietal (*r* = −0.02, *P* = 0.0002) and left paracentral (*r* = 0.02, *P* = 1.9e^−05^)—showing significantly smaller area in probable sarcopenia relative to non-sarcopenia (Supplementary Fig. 4; Supplementary Table 5).

For brain volumes (Fig. 2B; Supplementary Table 6), the analyses revealed significant group differences for 19 (i.e. 61.3%) of the 31 included structures. We observed the strongest effects for the lower brainstem (*r* = −0.05, *P* = 2.3e^−13^), cerebellum white matter (left/right *r* = −0.04, *P* = 2.7e^−15^/2.6e^−12^) and cerebellum cortex (left/right *r* = −0.04, *P* = 2.5e^−10^/1.2e^−13^) volumes in probable sarcopenia relative to non-sarcopenia.

For white matter FA, the analyses revealed widespread significantly lower FA in probable sarcopenia relative to non-sarcopenia in 37 (i.e. 77.1%) of the 48 included regions (Fig. 2C; Supplementary Table 7). We observed the strongest effects for brainstem tracts and commissural pathways, including the cerebral peduncle (CP, a projection fibre passing through the brainstem but sampled outside the brainstem:^36^ left/right *r* = −0.05/−0.06, *P* = 3.8e^−22^/4.2e^−29^), medial lemniscus (ML: left/right *r* = −0.05/−0.06, *P* = 2.5e^−22^/3.5e^−24^), superior cerebellar peduncle (SCP: left/right *r* = −0.05, *P* = 2.1e^−18^/8.6e^−20^) and the body and genu of the corpus callosum (BCC: *r* = −0.05, *P* = 1.5e^−16^; and GCC: *r* = −0.04, *P* = 3.9e^−16^).

For completeness, we performed sensitivity analyses to verify that the above results for probable sarcopenia were not driven by the participant subset with confirmed sarcopenia (*n* = 123). To this end, we first removed 163 (9.6%) of the participants (*n* = 123 with confirmed sarcopenia and *n* = 40 with probable sarcopenia and missing ALM) before repeating the same analyses with fewer participants in the probable sarcopenia group (*n* = 1541). The non-sarcopenia group (*n* = 32 171) remained the same. The sensitivity analyses suggest that the effect size pattern remains similar, albeit with slightly fewer significant regions (31.6%) across cortical thickness and area, brain volumes and microstructural white matter FA for the smaller and healthier probable sarcopenia sample than in the primary analyses. The significant range for *r* effect sizes remained similar, with |*r*| in (0.02, 0.06) and *P*-values in (0.0002, 2.2e^−26^) (Supplementary Tables 8–11; Supplementary Fig. 5).

### Brain structure and total thigh MFI

**Figure 3:**
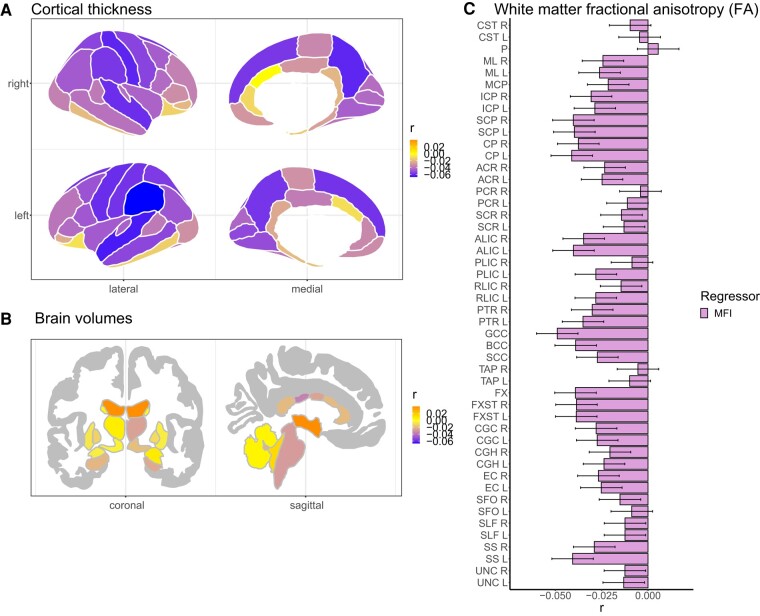
**Brain structure associations with total MFI.** We used multiple linear regression to obtain the partial correlation *r* effect size maps for total thigh MFI in percentage (*n* = 30 561) and (**A**) cortical thickness, (**B**) brain volumes and (**C**) white matter FA. For white matter FA, we additionally display the 95% confidence interval. Significant *r* effect sizes are |*r*| in (0.02, 0.07) and *P*-values in (0.0002, 1.9e^−31^). We adjusted for sex, age, age^2^, sex-by-age, sex-by-age^2^, body mass index, ancestry, metabolic/lifestyle variables, higher education, site, ICV (except FA and cortical thickness) and Euler numbers (T_1_-weighted MRI). FA, fractional anisotropy; ICV, estimated intracranial volume; L, left; R, right; *r*, partial correlation coefficient; *Brainstem tracts*: CST, corticospinal tract; ICP, inferior cerebellar peduncle; MCP, middle cerebellar peduncle; ML, medial lemniscus; P, pontine; SCP, superior cerebellar peduncle. *Projection pathways*: ACR, anterior corona radiata; ALIC, anterior limb of the internal capsule; CP, cerebral peduncle; PCR, posterior corona radiata; PLIC, posterior limb of the internal capsule; RLIC, retrolenticular part of the internal capsule; PTR, posterior thalamic radiation; SCR, superior corona radiata. *Commissural pathways*: BCC, body of corpus callosum; GCC, genu of corpus callosum; SCC, splenium of the corpus callosum; TAP, tapetum. *Association pathways:* CGC, cingulum cingulate gyrus; CGH, cingulum (hippocampal portion); EC, external capsule; FX, fornix; FXST, fornix stria terminalis; SFO, superior fronto-occipital fasciculus; SLF, superior longitudinal fasciculus; SS, sagittal stratum; UNC, uncinate fasciculus.

Overall, in the participants subsample with a total thigh MFI measure (*n* = 30 561) in percentage, we found *99* study-wide significant brain structural and microstructural features associated with MFI (Fig. 3; Supplementary Tables 12–15), amounting to 46.1% of the included brain regions from cortical thickness and area, brain volumes and microstructural white matter FA. The observed differences were small, with significant *r* effect sizes |*r*| in (0.02, 0.07) and *P*-values in (0.0002, 1.9e^−31^).

The analyses revealed a thinner cortex with higher MFI for 52 (76.5%) of the 68 cortical parcellations (Fig. 3A; Supplementary Table 12). We observed the strongest effects for the temporal [superior temporal (left/right *r* = −0.06, *P* = 2.1e^−28^/6.6e^−25^)], frontal (pre-central (left/right *r* = −0.06/*r* = −0.05, *P* = 4.3e^−25^/9.1e^−21^), superior frontal [left/right *r* = −0.06, *P* = 7.2e^−24^/3.4e^−23^)] and parietal [post-central (left/right *r* = −0.06, *P* = 6.2e^−24^/1.8e^−26^], supramarginal (left/right *r* = −0.07*/r* = −0.06, *P* = 1.9e^−31^/3.3e^−23^), precuneus (left/right *r* = −0.06, *P* = 1.4e^−23^/1.9e^−26^) and superior parietal [left/right *r* = −0.06/*r* = −0.05, *P* = 1.5e^−22^/1.5e^−20^)] lobes. There was limited evidence of associations between MFI and cortical area, with only six cortical parcellations showing significant effects (Supplementary Fig. 6; Supplementary Table 13).

For brain volumes, we observed significant associations with 11 (35.5%) of the included 31 brain volumes, with the strongest effects for lower mid-posterior corpus callosum (*r* = −0.04, *P* = 3.9e^−10^) and higher volumes of cerebrospinal fluid (*r* = 0.04, *P* = 4.5e^−13^), third ventricle (*r* = 0.04, *P* = 3.1e^−12^) and lateral ventricles (left/right *r* = 0.04, *P* = 9.5e^−12^/3.9e^−12^) at higher MFI (Fig. 3B; Supplementary Table 14).

For white matter FA, we observed significantly lower FA at higher MFI in 30 (62.5%) of the 48 included white matter regions (Fig. 3C; Supplementary Table 15), with the strongest effects for the GCC (*r* = −0.05, *P* = 1.1e^−17^), BCC (*r* = −0.04, *P* = 8.7e^−12^), fornix (*r* = −0.04, *P* = 7.9e^−12^), CP (left/right *r* = −0.04, *P* = 6.1e^−13^/4.8e^−11^) and SCP (left/right *r* = −0.04, *P* = 3.7e^−12^/1.9e^−12^).

### Brain structure mediates the link between sarcopenic and cognitive traits

**Figure 4:**
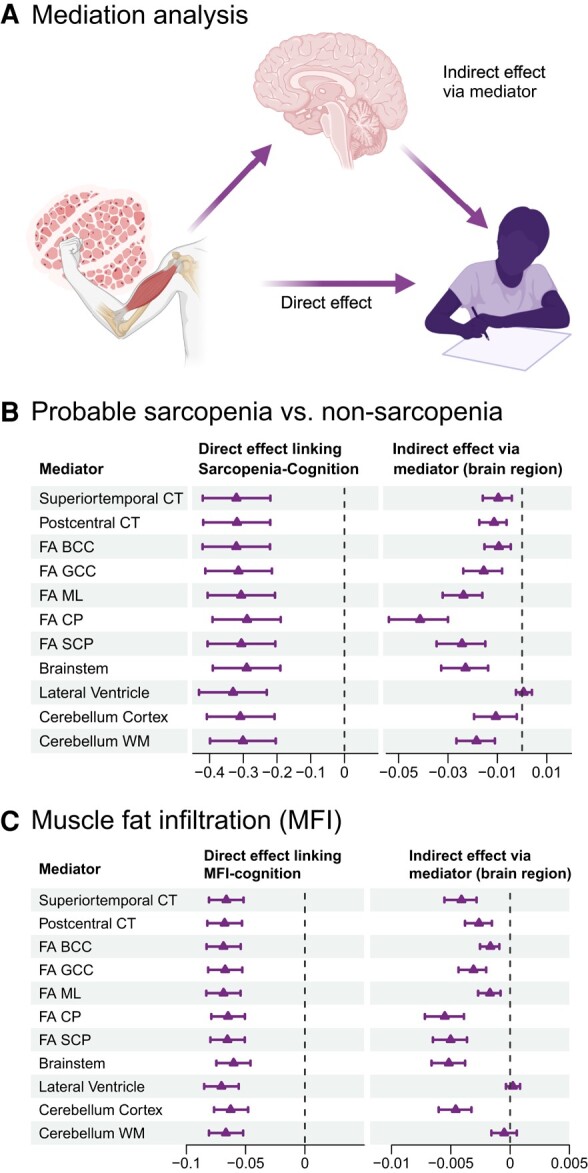
**Brain structure mediates the link between sarcopenic traits and cognitive performance.** The figure shows **(A)** an illustration of the mediation analysis (created with BioRender.com); and the results of the SEM mediation analyses for (**B**) probable sarcopenia (*n* = 1190) relative to non-sarcopenia (*n* = 21 340) with significant direct effects in (−0.33, −0.29) [*P*-values in (1.4e^−10^, 1.9e^−08^)] and indirect effects in (−0.04, −0.01) [*P*-values in (1.0e^−11^, 0.0001)]; and (**C**) continuously measured total thigh MFI in percentage (*n* = 20 850) with significant direct effects in (−0.07, −0.06) [*P*-values in (0, 2.2e^−16^)] and indirect effects in (−0.006, −0.002) [*P*-values in (1.2e^−12^, 7.7e^−05^)], in relation to cognitive performance (direct effect) and via mediator (brain phenotype; indirect effect). The direct and indirect effect estimates and corresponding confidence intervals are obtained from the SEM mediation model. We adjusted for sex, age, age^2^ body mass index, ancestry, metabolic/lifestyle variables, higher education, site, ICV (except FA and cortical thickness) and Euler numbers (T_1_-weighted MRI). BCC, body of corpus callosum; CP, cerebral peduncle; CT, cortical thickness; FA, fractional anisotropy; GCC, genu of corpus callosum; MFI, muscle fat infiltration; ML, medial lemniscus; SCP, superior cerebellar peduncle; WM, white matter.

We performed SEM mediation analyses (Fig. 4A) to investigate whether brain structure mediates the link between sarcopenic traits and general cognitive performance (PC1), focusing on the regions showing the largest effects in the primary analyses. We included the following regions: cortical thickness of the superior temporal and post-central gyri; volumes of the brainstem, cerebellum cortex, cerebellum white matter and lateral ventricle; and white matter FA of the CP, SCP, ML, BCC and GCC. We included the average measure for bilateral structures since we, in the primary analyses, observed similar findings for both sets of analyses with Pearson correlated coefficient of the left and right hemisphere *r* effect sizes equal to 0.99 and to limit the number of tests.

For probable sarcopenia (*n* = 1190) relative to non-sarcopenia (*n* = 21 340), the mediation analysis showed significant *direct effects* with lower general cognitive performance (PC1) with estimates in (−0.29, −0.33), *z*-scores in (−6.42, −5.63) and *P*-values in (1.3e^−08^, 4.54e^−10^) (Fig. 4B; Supplementary Table 16), similar to the primary analyses. We found significant *indirect effects* (i.e. mediation effect) for several brain phenotypes, including the thickness of the post-central gyrus (estimate = −0.01, *z*-score = −4.01, *P* = 6.0e^−05^), brainstem (estimate = −0.02, *z*-score = −4.74, *P* = 2.2e^−06^) and cerebellum white matter (estimate = −0.02, *z*-score = −4.58, *P* = 4.6e^−06^) volumes and the FA of the GCC (estimate = −0.02, *z*-score = −3.87, *P* = 0.0001), ML (estimate = −0.02, *z*-score = −5.71, *P* = 1.1e^−08^), CP (estimate = −0.04, *z*-score = −6.80, *P* = 1.0e^−11^) and SCP (estimate = −0.02, *z*-score = −4.86, *P* = 1.2e^−06^). We observed the most significant mediation effects for the FA of the included brainstem white matter tracts. For the CP, which shows the greatest effect size, the indirect effect was more significant than the direct effect.

Amongst the participants with measures of total thigh MFI in percentage and PCI (*n* = 20 850), SEM mediation analyses revealed a significant *direct effect* between higher MFI and lower general cognitive performance **(**PC1), with estimates in (−0.06, −0.07), *z*-scores in (−8.16, −9.49) and *P*-values in (0, 2.2e^−16^) (Fig. 4C; Supplementary Table 17**)**. We found significant *indirect effects* for the thickness of the superior temporal (estimate = −0.004, *z*-score = −5.94, *P* = 2.9e^−09^) and post-central (estimate = −0.002, *z*-score = −4.56, *P* = 5.1e^−06^) gyri, volumes of the brainstem (estimate *=*  *−0.005*, *z*-score = −7.11, *P* = 1.7e^−12^) and cerebellum cortex (estimate = −0.005, *z*-score = −6.54, *P* = 6.2e^−11^) and FA of the BCC (estimate = −0.002, *z*-score = −3.95, *P* = 7.7e^−05^), GCC (estimate = −0.003, *z*-score = −5.22, *P* = *−*1.8e^−07^) and CP (estimate = −0.006, *z*-score = −6.53, *P* = 6.6e^−11^). We observed the most significant indirect effects via the brainstem, followed by the cerebellum cortex, CP and superior temporal gyrus.

For both sets of SEM mediation analyses, probable sarcopenia versus non-sarcopenia and the continuous MFI measurement, the total effect (i.e. the added effect of the direct and indirect effect via brain structure) was generally lower and more significant than the direct effect for all included brain structures (except lateral ventricle for MFI; Supplementary Tables 16 and 17). These findings suggest that brain structures contribute to the observed associations between the two sarcopenic traits and general cognitive performance.

## Discussion

In the current study of a middle-aged and older adult sample, we show significant associations between two sarcopenic traits (probable sarcopenia versus non-sarcopenia and high MFI) and lower general cognitive performance and various brain structural grey and white matter features. SEM analysis further demonstrated that brain structures involved in the sensorimotor system mediate the relationship between sarcopenic and cognitive traits. Our results support the notion of an interplay between muscles and the brain and suggest links between sarcopenic traits (low muscular strength and quality) and cognitive decline, which may be relevant for the development of dementia.

Our results show that brain phenotypes of mainly the sensorimotor system exhibited the largest effect sizes of the identified group differences in probable sarcopenia relative to non-sarcopenia; this was true for the cortex, white matter FA, cerebellum and brainstem. Our analysis further suggested a mediating role for specific brain phenotypes of the sensorimotor system between sarcopenic and cognitive traits. Additionally, we observed widespread group differences, most extensively for white matter FA, followed by brain volumes and cortical thickness. Thus, our findings suggest that the brain sensorimotor system is involved in the development of sarcopenia-associated cognitive dysfunction. Our results are in line with a recent review indicating links between sensory impairment and sarcopenia and sarcopenic traits, including lower muscular mass and physical performance,^55^ while loss of motor function is an aspect of sarcopenia. Indeed, sensory input and motor command are clinically relevant assessments of muscle coordination and function. Skeletal muscle function relies on preserved innervation.^56^ A gradual deterioration of muscles and sensory nerve action potentials with age is reported.^57^ Conduction speed reduction is secondary to reduced myelination and functional loss of the largest axonal fibers.^58^ These age-related changes in the conduction velocities are associated with loss of muscular strength.^58,59^ Here, we show that brain structural variations in the sensorimotor system—including the thickness of post-central and superior temporal gyri and structure and microstructure of the cerebellum and brainstem—are associated with probable sarcopenia and that they mediate the link between probable sarcopenia and cognitive performance. Although causal interpretations remain speculative, it is possible that abnormal sensory functioning makes people more prone to develop sarcopenia and cognitive decline.

A feature of muscle loss and sarcopenia development is the accumulation of ectopic muscle fat (i.e. MFI). We observed that a higher percentage of MFI was associated with a thinner cortex across the majority of cortical regions, with the most significant findings for temporal, frontal and parietal lobes, extending our previous results of cortical thinning at higher MFI using a partly overlapping smaller sample.^15^ Notably, the pattern observed in this study is more pronounced and widespread, most likely due to the larger sample. Additionally, we showed that higher MFI is associated with widespread white matter FA and brain volume phenotypes but with less significant effect sizes than cortical thickness. The largest effect sizes for MFI were more significant than those observed for probable sarcopenia relative to non-sarcopenia. Although MFI is a component of sarcopenia, high MFI appears as a separate factor more strongly linked to cortical thinning than probable sarcopenia. Prior studies indicate that ectopic muscle fat accumulation is associated with cardiometabolic risk factors, disease outcomes and mortality,^17,60,61^ and high MFI may represent ectopic fat accumulation linked to abdominal fat, obesity and cardiometabolic disturbance. Indeed, we showed that higher MFI is associated with lower cognitive performance. The skeletal muscle and body fat tissue are insulin sensitive,^62^ and a recent review further suggested an interplay between obesity and insulin disturbance with links to cognitive dysfunction,^63^ potentially through metabolic hormones and modulation of plasticity.^64^ There is growing evidence linking obesity and insulin resistance to cognitive impairment and Alzheimer’s disease.^65,66^ Thus, elevated MFI may be relevant for the development of cognitive and brain-related traits and disorders.

The greater effect sizes and more extensive effects observed for white matter FA than grey matter structure in probable sarcopenia relative to non-sarcopenia may suggest that aberrations in white matter FA precede grey matter alterations, possibly relating to, e.g. inflammatory processes, demyelination and axonal degeneration as previously suggested for white matter lesions and dementia-related white matter alterations.^67-69^ Although cortical thinning was the most pronounced brain feature associated with higher MFI, we also here observed widespread associations with lower white matter FA and brain volumes (e.g. cerebellum). Thus, although the extent of the effect size and significance patterns differed somewhat, both probable sarcopenia and MFI were associated with widespread cortical thinning, lower white matter FA and brain volume alterations, and brain structures of the sensorimotor system mediated the link between both sarcopenic traits and general cognitive performance. Thus, our findings provide important potential cues to understand the relationship between muscle and brain morphological disturbances in relation to the development of sarcopenia and dementia.

Our study provides evidence of close connections between sarcopenic traits, brain structure and cognitive performance, suggesting similar underlying mechanisms for sarcopenic and cognitive impairments mediated by brain structure. Indeed, shared mechanisms may cause people with cognitive decline and sarcopenia to have more severe and widespread brain structure abnormalities. However, a lack of muscular activity might also lead to neurodegeneration or vice versa. Multiple factors may be involved, including sedentarism, malnutrition, little sensory stimuli^2,5^ and inflammation,^70,71^ which all may work together in a vicious circle and accelerate the degenerative neuronal processes. Degenerative processes also affect white matter microstructure,^67-69^ and neurodegeneration in the central nervous system can affect peripheral functions through several mechanisms, including mitochondrial deficiency, peripheral neuronal degeneration, abnormalities in the neuromuscular junctions and slowness of peripheral motor nerve velocity.^72,73^ The current findings may suggest that brain-related neurodegenerative processes are related to muscle malfunction as manifested by sarcopenic traits in parallel with cognitive decline.

Due to the demographic transition, the proportion of older adults is increasing worldwide, and despite fewer age-related dementia cases, there are an increasing number of people living with dementia.^74,75^ Dementia represents a significant burden due to its severe consequences for the affected individuals and the societal costs.^74^ It is a multifactorial complex condition,^76^ and developing intervention strategies is critical.^75,77^ A recent study identified modifiable risk and protective factors for dementia related to lifestyle, medical history, socioeconomic status and physical measures.^78^ Markers of early dementia include cognitive impairment,^76^ brain imaging abnormalities^79^ and impaired handgrip strength.^80^ Our study connects sarcopenic traits, brain grey and white matter phenotypes and general cognitive performance. Although the causal directionality of (possibly reciprocal) effects remains elusive, our findings are in line with the hypothesis that physical activity enhances brain health and cognitive functioning.

The current study offers many strengths, including the unprecedented sample size, well-characterized data and an automated quality control and analysis pipeline. The results address relevant gaps in the scientific literature and provide new insights into a possible central mechanism associated with the relationship between sarcopenic traits, the brain’s grey and white matter structure and cognition, which may be relevant to clinical care. Our findings are all of small effects, which is in line with other brain imaging research,^81,82^ and of polygenic architecture in dementia.^83^ Indeed, small effects can be robustly captured only by larger studies with adequate power to identify true effects,^81^ as we did in the current study. The study also has some limitations. We did not exclude participants based on neurological disease and other comorbidities of sarcopenia, which may influence the findings, and we did not investigate the role of comorbid conditions in the observed cognitive and brain phenotype findings. We limited the mediation analyses to the most significant findings from the primary analyses, which may influence our results, and future studies should replicate the findings in independent samples. Additionally, we adjusted the statistical models for relevant cardiometabolic risk factors and educational attainment, but not all dementia-specific risk factors.^75^ We did not adjust for physical activity or exercise, which may be protective factors for the development and progression of sarcopenia and cognitive impairments, and these aspects should be addressed in future work. Due to the data availability in the UK Biobank, we did not assess and adjust for frailty or other related comorbidities^1,84^ of sarcopenia. Although muscle quantity or quality assessment from whole-body MRI is considered optimal,^1^ we used ALM from bioimpedance to confirm sarcopenia diagnosis due to a lack of valid European Working Group on Sarcopenia in Older People cut-offs for muscle quantity or quality from MRI. At the group level, the UK Biobank participants are healthier than the general population,^85^ the imaging sub-sample is even healthier,^86^ and the participant sample is predominantly of white European ancestry. Thus, our sample reflects a subset of the general population. The UK Biobank deployed a cognitive test battery explicitly developed for population-based cognitive testing,^22^ which reportedly is moderate to highly correlated with standardized psychometric tests.^23^ Further studies are needed to understand the complex, possibly reciprocal, mechanisms that can co-occur, such as inflammation.

## Conclusion

In conclusion, the current findings demonstrate that sarcopenic traits are associated with cognitive performance and widespread brain phenotypes, including cortical thickness, brain volumes and white matter microstructure. Furthermore, brain structures involved in the sensorimotor system mediated the association between sarcopenic and cognitive traits. These findings may be important for understanding the development of sarcopenia and cognitive impairments and the sarcopenia–dementia link and support further development of intervention strategies for treating degenerative processes affecting both the body and the brain.

## Supplementary Material

fcae083_Supplementary_Data

## Data Availability

The UK Biobank resource is open for eligible researchers upon application (http://www.ukbiobank.ac.uk/register-apply/). We extracted data from the UK biobank baskets using the *ukb_helper.py* script (https://github.com/precimed/ukb). We used publicly available resources to process the brain image data and conduct statistical analyses. We have made the project R-scripts publicly available at https://doi.org/10.17605/OSF.IO/TXBWA.
