# Supplementary material for: Linking sarcopenia, brain structure and cognitive performance: a large-scale UK Biobank study
Source: Brain Commun. 2024 Mar 7;6(2):fcae083. doi: 10.1093/braincomms/fcae083 (PMC10953622; doi:10.1093/braincomms/fcae083)
Supplement: fcae083_Supplementary_Data [file fcae083_supplementary_data.pdf]

Linking Sarcopenia, Brain Structure, and Cognitive Performance: A large-scale UK Biobank study.

Supplementary Material

Tiril P. Gurholt[1], Miguel Germán Borda[2,3,4], Nadine Parker[1], Vera Fominykh[1], Rikka Kjelkenes[1,5], Jennifer Linge[6,7], Dennis van der Meer[1,8], Ida E. Sønderby[1,9], Gustavo Duque[10], Lars T Westlye[1,5], Dag Aarsland[2,11], Ole A Andreassen[1]

Author affiliations:

[1] Norwegian Centre for Mental Disorders Research (NORMENT), Division of Mental Health and Addiction, Oslo University Hospital and University of Oslo, 0424 Oslo, Norway

[2] Centre for Age-Related Medicine (SESAM), Stavanger University Hospital, 4068 Stavanger, Norway

[3] Faculty of Health Sciences, University of Stavanger, 4036 Stavanger, Norway.

[4] Semillero de Neurociencias y Envejecimiento, Ageing Institute, Medical School, Pontificia Universidad Javeriana, 111611 Bogotá, Colombia

[5] Department of Psychology, University of Oslo, 0373 Oslo, Norway

[6] AMRA Medical AB, 58222 Linköping, Sweden

[7] Department of Health, Medicine and Caring Sciences, Linköping University, 58183 Linköping, Sweden

[8] School of Mental Health and Neuroscience, Faculty of Health, Medicine and Life Sciences, Maastricht University, 6200MD Maastricht, The Netherlands

[9] Department of Medical Genetics, Oslo University Hospital, 0424 Oslo, Norway

[10] Dr. Joseph Kaufmann Chair in Geriatric Medicine, Department of Medicine and Research Institute of the McGill University Health Centre, McGill University, H4A 3J1 Montreal, QC, Canada

[11] Department of Psychological Medicine, Institute of Psychiatry, Psychology, and Neuroscience, King's College London, WC2R 2LS London, UK

Correspondence to*:* Tiril P Gurholt, Ph.D.

Division of Mental Health and Addiction, Oslo University Hospital, P.O. Box 4956 Nydalen, 0424 OSLO, Norway.

E-mail: t.p.gurholt@ous-research.no.

Table of contents

Supplementary Tables 5

Supplementary Table 1: Demographics and clinical data - split on sex. 5

Supplementary Table 2: Demographics and clinical data for probable sarcopenia vs non-sarcopenia 7

Supplementary Table 3: Overview of the included diffusion-MRI regions of interest. 9

Supplementary Table 4: Cortical thickness alterations in probable sarcopenia relative to non-sarcopenia. 10

Supplementary Table 5: Cortical area alterations in probable sarcopenia relative to non-sarcopenia. 12

Supplementary Table 6: Brain volume alterations in probable sarcopenia relative to non-sarcopenia. 14

Supplementary Table 7: White matter microstructure (fractional anisotropy) alterations in probable sarcopenia relative to non-sarcopenia. 15

Supplementary Table 8: Cortical thickness alterations in probable sarcopenia relative to non-sarcopenia after removing participants with confirmed sarcopenia. 16

Supplementary Table 9: Cortical area alterations in probable sarcopenia relative to non-sarcopenia after removing participants with confirmed sarcopenia. 17

Supplementary Table 10: Brain volume alterations in probable sarcopenia relative to non-sarcopenia after removing participants with confirmed sarcopenia. 18

Supplementary Table 11: White matter (fractional anisotropy) alterations in probable sarcopenia relative to non-sarcopenia after removing participants with confirmed sarcopenia. 19

Supplementary Table 12: The association between cortical thickness and muscle fat infiltration (MFI). 20

Supplementary Table 13: The association between cortical area and muscle fat infiltration (MFI). 22

Supplementary Table 14: The association between brain volumes and muscle fat infiltration (MFI). 24

Supplementary Table 15: The association between white matter microstructure (fractional anisotropy) and muscle fat infiltration (MFI). 25

Supplementary Table 16: The results of the mediation analyses for probable sarcopenia relative to non-sarcopenia. 26

Supplementary Table 17: The results of the mediation analyses for muscle fat infiltration (MFI). 27

Supplementary Figures 28

Supplementary Figure 1: Overview of the included cortical parcellations. 28

Supplementary Figure 2: Overview of the included deep and lower brain structures. 29

Supplementary Figure 3: Principal component analysis across cognitive test statistics. 30

Supplementary Figure 4: Probable sarcopenia vs. non-sarcopenia on the cortical area. 31

Supplementary Figure 5: Sensitivity analyses for probable sarcopenia vs. non-sarcopenia after removing the confirmed sarcopenia participants. 32

Supplementary Figure 6: Total muscle fat infiltration on the cortical area. 34

Supplementary Notes 35

Supplementary Note 1: Extracted UK Biobank Field-IDs 35

Supplementary Table N1: Overview of extracted UK Biobank data Field-IDs 36

Supplementary Table N2: The Included cognitive tests. 37

Supplementary Note 2: Details of UK Biobank Assessment 38

Handgrip Strength 38

Whole-Body Bioimpedance 38

Cognitive Test Battery 38

Supplementary Note 3: Estimate Appendicular Lean Mass 39

Supplementary Note 4: Diffusion MRI Processing 40

Supplementary references 41

Supplementary Tables

Supplementary Table 1: Demographics and clinical data - split on sex.

Variables	Men	Women	test	p-value
**N**	15424	18285		
**Age (year)**[1]	63.7±7.5	62.7±7.3	12.5	1.38632023104452e−35
**Age range (year)**	[44 82]	[45 81]		
**European**[2]	14932 (96.8)	17773 (97.2)	4.3	0.03893578561798
**Higher Education**[2]	9842 (63.8)	11520 (63)	2.3	0.128229570816099
**Probable sarcopenia**[2]	746 (4.8)	957 (5.2)	2.7	0.102300833660117
**Maximum handgrip (kg)**[1]	40.3±8.5	24.8±6	190.8	0
**ALM (kg)**[1,3]	25.6±3.7	17.7±2.2	232.9	0
**ALM (kg/height^2)**[1,3]	8.2±1	6.7±0.8	159.6	0
**Unable/Slow/Average/Brisk walking pace**[4]	21/545/7612/7279	24/831/8899/8564		
**No/One/More falls**[5]	13444/1474/530	14416/2832/1075		
**Duration of walks (minutes)**[1,6]	57.8±64	60.8±67	−4	5.85385845016785e-05
**Duration of moderate activity (minutes)**[1,7]	63.2±67.4	63.7±62.2	-0.7	0.47915131732023
**Duration of vigorous activity (minutes)**[1,8]	44.9±42.5	41.2±36.6	6.9	5.17974968396892e-12
**Total MFI (%)**[1,9]	8.4±1.9	9.6±2	−54	0
**Anterior MFI (%)**[1,10]	6.6±1.6	7.7±1.8	−55.4	0
**Posterior MFI (%)**[1,11]	10.1±2.2	11.4±2.3	−50.5	0
**Smoker**[2]	617 (4)	526 (2.9)	31.9	1.62220558908068e−08
**Smoker Current/previous/never**	617 / 5532 / 9275	526 / 5581 / 12178		
**Alcohol drinker**[2]	14587 (94.6)	16902 (92.4)	61.8	3.88463534137054e−15
**Alcohol drinker Current/previous/never**	14587 / 488 / 349	16902 / 634 / 749		
**Height (cm)**[1]	176.3±6.6	162.9±6.2	189.8	0
**Weight (kg)**[1]	83.3±13.2	68.9±13	100.6	0
**BMI**[1]	26.8±3.8	26±4.7	17.4	1.2315456757795e−67
**Waist circumference (cm)**[1,12]	93.6±10.4	82.5±11.6	92.5	0
**Hip circumference (cm)**[1,9]	100.5±7.2	100.8±9.7	−2.6	0.00975151892029438
**WHR**[1,13]	0.9±0.1	0.8±0.1	157.1	0
**Diabetic**[2]	367 (2.4)	208 (1.1)	76.2	2.54086029110122e−18
**High cholesterol**[2]	2378 (15.4)	1679 (9.2)	306.6	1.17407677805459e−68

[1] Welch Two Sample t-test
[2] Pearson's Chi-squared test with Yates' continuity correction
[3] Missingness: women n=366, men n=283
[4] Missingness: women n=11, men n=11
[5] Missingness: women n=19, men n=12
[6] Missingness: women n=1,878, men n=938
[7] Missingness: women n=2,982, men n=1,982
[8] Missingness: women n=6,981, men n=4,682
[9] Missingness: women n=1,582, men n=1,566
[10] Missingness: women n=1,579, men n=1,564
[11] Missingness: women n=1,578, men n=1,532
[12] Missingness: women n=2, men n=0
[13] Missingness: women n=3, men n=0

**Hypertension**[2]	3681 (23.9)	3001 (16.4)	291.9	1.87195393441935e−65
**Neurological condition**	54 (0.4)	98 (0.5)	6	0.0140589340790113

Notes: The data on ethnic ancestry, number of falls, activity levels (walking speed, duration of walks, moderate and vigorous activity), smoking, alcohol consumption, and diagnostic data was self-reported. *Abbreviations:* BMI – body mass index; MFI – muscle fat infiltration; WHR – waist-to-hip ratio.

Supplementary Table 2: Demographics and clinical data for probable sarcopenia vs non-sarcopenia

Variables	Probable sarcopenia	Non-sarcopenia	test	p-value
**N**	1703	32006		
**Women**[1]	957 (56.2)	17328 (54.1)	2.7	0.102300833660117
**Age (year)**[2]	66.9±7.1	62.9±7.4	22.5	2.38449032143083e-99
**Age range (year)**	[45 81]	[44 82]		
**European**[1]	1634 (95.9)	31071 (97.1)	6.8	0.00930386056491692
**Higher Education**[1]	972 (57.1)	20390 (63.7)	30.3	3.6154755010176e-08
**Maximum Handgrip (kg)**[2]	17.1±6	32.7±10.2	-100.1	0
**Maximum handgrip (kg, range)**	[1 26]	[16 90]		
**ALM (kg)** [2,3]	20.1±4.5	21.4±5	-11.8	7.66045402328449e-31
**ALM (kg/height^2)**[3,4]	7.3±1.2	7.4±1.2	-3.3	0.00105065237341942
**Unable/Slow/Average/Brisk walking pace**[5]	10 / 206 / 944 / 555	35 / 1170 / 15567 / 15288		
**No/One/More falls**[6]	1283 / 267 / 157	26577 / 4039 / 1448		
**Duration of walks (minutes)**[4,7]	56.3±64	59.6±65.7	-1.9	0.0592544513696296
**Duration of moderate activity (minutes)**[2,8]	65.5±68.7	63.4±64.4	1.1	0.257863056182997
**Duration of vigorous activity (minutes)**[2,9]	40.3±37.5	43.1±39.7	-2.2	0.0272435184449438
**Total MFI (%)**[2,10]	9.8±2.1	9±2	13.8	2.23356525601911e-41
**Anterior MFI (%)**[2,11]	8±2	7.2±1.8	14.3	4.18139453297019e-44
**Posterior MFI (%)**[4,12]	11.6±2.4	10.8±2.3	12.9	3.0688072871514e-38
**Smoker**[1]	38 (2.2)	1105 (3.5)	7	0.00818607796726802
**Smoker Current/previous/never**	38 / 573 / 1092	1105 / 10540 / 20361		
**Alcohol drinker**[1]	1526 (89.6)	29963 (93.6)	41.6	1.10873014779916e-10
**Alcohol drinker Current/previous/never**	1526 / 88 / 89	29963 / 1034 / 1009		
**Height (cm)** [2]	165.1±8.9	169.2±9.2	-18.5	9.96301512319444e-71
**Weight (kg)** [2]	72.8±14.2	75.7±14.9	-8.1	8.7632274775703e-16
**BMI**[2]	26.7±4.6	26.3±4.3	2.8	0.00595513645679748
**Waist circumference (cm)**[4,13]	88.3±12.5	87.5±12.4	2.6	0.00950172549015854
**Hip circumference (cm)** [2,10]	100.2±9	100.7±8.6	-2.1	0.0396659088448594
**WHR**[4,14]	0.9±0.1	0.9±0.1	5.7	1.20590207408748e-08
**Diabetic**[1]	48 (2.8)	527 (1.6)	12.6	0.000394806462177617
**High cholesterol**[1]	326 (19.1)	3731 (11.7)	84.9	3.17860103598448e-20
**Hypertension**[1]	422 (24.8)	6260 (19.6)	27.4	1.65003389840061e-07
**Neurological condition**[1]	17 (1)	135 (0.4)	10.7	0.00105994564888933

[1] Pearson's Chi-squared test with Yates' continuity correction
[2] Welch Two Sample t-test
[3] Missing: probable sarcopenia n=40, non-sarcopenia n=609
[4] Two Sample t-test
[5] Missing: probable sarcopenia n=4, non-sarcopenia n=18
[6] Missing: probable sarcopenia n=2, non-sarcopenia n=29
[7] Missing: probable sarcopenia n=211, non-sarcopenia n=2,605
[8] Missing: probable sarcopenia n=357, non-sarcopenia n=4,607
[9] Missing: probable sarcopenia n=805, non-sarcopenia n=10,858
[10] Missing: probable sarcopenia n=163, non-sarcopenia n=2985
[11] Missing: probable sarcopenia n=163, non-sarcopenia n=2980
[12] Missing: probable sarcopenia n=160, non-sarcopenia n=2950
[13] Missing: probable sarcopenia n=0, non-sarcopenia n=2
[14] Missing: probable sarcopenia n=0, non-sarcopenia n=3

Notes: The data on ethnic ancestry, number of falls, activity levels (walking speed, duration of walks, moderate and vigorous activity), smoking, alcohol consumption, and diagnostic data was self-reported. *Abbreviations:* BMI – body mass index; MFI – muscle fat infiltration; WHR – waist-to-hip ratio.

Supplementary Table 3: Overview of the included diffusion-MRI regions of interest.

Abbreviation	Full name	Abbreviation	Full name
**ACR**[*]	Anterior corona radiata	**RLIC**[*]	Retrolenticular part of IC
**ALIC**[*]	Anterior limb of the internal capsule	**SCC**	Splenium of the corpus callosum
**BCC**	Body of corpus callosum	**SCR**[*]	Superior corona radiata
**CGC**[*]	Cingulum cingulate gyrus	**SFO**[*]	Superior fronto-occipital fasciculus
**CGH**[*]	Cingulum (hippocampal portion)	**SLF**[*]	Superior longitudinal fasciculus
**CST**[*]	Corticospinal tract	**SS**[*]	Sagittal stratum
**CP***	Cerebral peduncle	**UNC**[*]	Uncinate fasciculus
**ML***	Medial lemniscus		
**ICP***	Inferior cerebellar peduncle		
**MCP**	Middle cerebellar peduncle		
**SCP***	Superior cerebellar peduncle		
**P**	Pontine		
**EC**[*]	External capsule		
**FX**	Fornix		
**FXST***	Fornix stria terminalis		
**T***	Tapetum		
**GCC**	Genu of corpus callosum		
**PCR**[*]	Posterior corona radiata		
**PLIC**[*]	Posterior limb of the internal capsule		
**PTR**[*]	Posterior thalamic radiation		

***** For bilateral structures, we include left and right hemisphere measures

Supplementary Table 4: Cortical thickness alterations in probable sarcopenia relative to non-sarcopenia.

**Left hemisphere**			**Right hemisphere**		
**Structure**	**r**	**p-value**	**Structure**	**r**	**p-value**
**lh bankssts**	-0.02	0.00040204560227096	**rh bankssts**	-0.02	**9.20393204208854e-06**
**lh caudalanteriorcingulate**	0.01	0.104296351282438	**rh caudalanteriorcingulate**	0.01	0.095036207297355
**lh caudalmiddlefrontal**	-0.02	0.00104837831951284	**rh caudalmiddlefrontal**	-0.01	0.00940463325811759
**lh cuneus**	-0.02	0.00380862091203431	**rh cuneus**	-0.02	0.00180645336208617
**lh entorhinal**	0	0.792518766834793	**rh entorhinal**	-0.01	0.278834763602673
**lh fusiform**	-0.03	**3.60276178411116e-06**	**rh fusiform**	-0.02	**5.48587945221333e-05**
**lh inferiorparietal**	-0.03	**8.64152496917132e-07**	**rh inferiorparietal**	-0.03	**1.36254734273018e-06**
**lh inferiortemporal**	0	0.766543275565454	**rh inferiortemporal**	-0.01	0.258200120964886
**lh isthmuscingulate**	0.01	0.0753540023474619	**rh isthmuscingulate**	0.01	0.191011427827249
**lh lateraloccipital**	-0.02	**0.000155915327508819**	**rh lateraloccipital**	-0.02	0.000301882884599843
**lh lateralorbitofrontal**	0.01	0.00674444834155692	**rh lateralorbitofrontal**	0.01	0.0197314627172318
**lh lingual**	-0.03	**9.47068469104193e-09**	**rh lingual**	-0.02	1.82236342243425e-05
**lh medialorbitofrontal**	0.01	0.0621669584020528	**rh medialorbitofrontal**	0.01	0.0185707865349171
**lh middletemporal**	-0.02	0.00444664187388661	**rh middletemporal**	-0.03	**3.08755111232307e-08**
**lh parahippocampal**	-0.01	0.0544199923345602	**rh parahippocampal**	-0.01	0.11683846474538
**lh paracentral**	-0.02	0.000622680325189885	**rh paracentral**	-0.02	0.00546936006255901
**lh parsopercularis**	-0.03	**3.01815705783197e-08**	**rh parsopercularis**	-0.02	9.51703046129111e-06
**lh parsorbitalis**	-0.01	0.0334305102851988	**rh parsorbitalis**	0	0.621554578597711
**lh parstriangularis**	-0.02	0.000226061100873103	**rh parstriangularis**	-0.02	**6.77624294138581e-05**
**lh pericalcarine**	-0.03	**3.95908886170706e-08**	**rh pericalcarine**	-0.03	**8.95224580913649e-10**
**lh postcentral**	-0.04	**2.2284551476915e-13**	**rh postcentral**	-0.04	**6.94834310957565e-14**
**lh posteriorcingulate**	0.01	0.200900573340754	**rh posteriorcingulate**	0.01	0.0318140671849614
**lh precentral**	-0.02	0.000300516279019	**rh precentral**	-0.03	**1.8087077399479e-06**
**lh precuneus**	-0.03	**4.93982081589924e-09**	**rh precuneus**	-0.03	**2.10299555170032e-09**
**lh rostralanteriorcingulate**	0.01	0.0339633686453251	**rh rostralanteriorcingulate**	0.01	0.0319599697512527
**lh rostralmiddlefrontal**	-0.02	0.000464112260484959	**rh rostralmiddlefrontal**	-0.01	0.0200029512070208
**lh superiorfrontal**	-0.01	0.0238376012870462	**rh superiorfrontal**	0	0.501210955037137
**lh superiorparietal**	-0.02	**5.90450991568998e-06**	**rh superiorparietal**	-0.03	**3.57591339770136e-06**
**lh superiortemporal**	-0.04	**7.78950011381719e-11**	**rh superiortemporal**	-0.04	**5.77806765516719e-11**
**lh supramarginal**	-0.02	**2.475377681518e-05**	**rh supramarginal**	-0.03	**2.01447711638165e-06**
**lh frontalpole**	0	0.569628765053021	**rh frontalpole**	0.01	0.0632787284647555
**lh temporalpole**	-0.01	0.0739627508768291	**rh temporalpole**	-0.01	0.0210422288593905
**lh transversetemporal**	-0.03	**7.43634944273623e-07**	**rh transversetemporal**	-0.02	**8.10808331815107e-05**
**lh insula**	-0.01	0.0654592205769838	**rh insula**	0	0.912698598295531

Notes: Significant p-values of structures indicated in bold. We adjust for sex, age, age^2, sex-by-age, sex-by-age^2, body mass index, ancestry, metabolic/lifestyle variables, higher education, site, and Euler numbers. *Abbreviations:* lh – left hemisphere; r - partial correlation coefficient; rh – right hemisphere.

Supplementary Table 5: Cortical area alterations in probable sarcopenia relative to non-sarcopenia.

**Left hemisphere**			**Right hemisphere**		
**Structure**	**r**	**p-value**	**Structure**	**r**	**p-value**
**lh bankssts**	-0.02	0.00244839903222396	**rh bankssts**	-0.01	0.132202623621434
**lh caudalanteriorcingulate**	0	0.883258831164059	**rh caudalanteriorcingulate**	0	0.989321345662411
**lh caudalmiddlefrontal**	0.02	0.00568855771060179	**rh caudalmiddlefrontal**	0.01	0.0161512807910367
**lh cuneus**	0	0.38812994385954	**rh cuneus**	0	0.929692749932589
**lh entorhinal**	-0.02	0.00048301479350108	**rh entorhinal**	-0.02	0.000902247469815916
**lh fusiform**	-0.02	**9.23926728251793e-05**	**rh fusiform**	-0.02	0.000765047858558848
**lh inferiorparietal**	-0.02	**0.000186121254045437**	**rh inferiorparietal**	-0.01	0.00870902564513293
**lh inferiortemporal**	-0.02	0.0053772085911703	**rh inferiortemporal**	-0.02	0.0023104077819226
**lh isthmuscingulate**	0.01	0.214750816227058	**rh isthmuscingulate**	0	0.883048436077733
**lh lateraloccipital**	0.01	0.221023150697841	**rh lateraloccipital**	0	0.99452774243167
**lh lateralorbitofrontal**	-0.02	0.00298802751860971	**rh lateralorbitofrontal**	-0.01	0.0891482673316139
**lh lingual**	-0.01	0.0084219298397541	**rh lingual**	-0.01	0.0355552270794884
**lh medialorbitofrontal**	-0.01	0.213441781946121	**rh medialorbitofrontal**	-0.01	0.152903629023834
**lh middletemporal**	-0.02	0.00262223413409189	**rh middletemporal**	-0.02	0.00200044038618816
**lh parahippocampal**	-0.01	0.166902679814014	**rh parahippocampal**	-0.01	0.226271928837735
**lh paracentral**	0.02	**1.89665803497073e-05**	**rh paracentral**	0.02	0.00129411622014468
**lh parsopercularis**	0	0.817029955143067	**rh parsopercularis**	0.01	0.238032412638811
**lh parsorbitalis**	-0.01	0.190474996785788	**rh parsorbitalis**	0	0.57911660194821
**lh parstriangularis**	0	0.816761733630771	**rh parstriangularis**	0	0.794624181232471
**lh pericalcarine**	-0.01	0.0460778733023493	**rh pericalcarine**	-0.01	0.0146635030465189
**lh postcentral**	0	0.73898498753953	**rh postcentral**	0	0.667178572119738
**lh posteriorcingulate**	0	0.489698332932584	**rh posteriorcingulate**	0	0.858722133802638
**lh precentral**	0.01	0.239985813100437	**rh precentral**	0.01	0.193305425915722
**lh precuneus**	0	0.517977427031281	**rh precuneus**	0	0.954896579998926
**lh rostralanteriorcingulate**	0	0.820660052683155	**rh rostralanteriorcingulate**	0	0.433295705103436
**lh rostralmiddlefrontal**	0.01	0.0230522608700447	**rh rostralmiddlefrontal**	0.01	0.0870796958225416
**lh superiorfrontal**	0.01	0.233036704971574	**rh superiorfrontal**	0.01	0.180229142976636
**lh superiorparietal**	0	0.817384064438198	**rh superiorparietal**	0	0.707134596102912
**lh superiortemporal**	0	0.647539762275221	**rh superiortemporal**	0	0.982365258356903
**lh supramarginal**	-0.01	0.128291592706023	**rh supramarginal**	-0.01	0.124599123288329
**lh frontalpole**	0	0.362518844123618	**rh frontalpole**	0.01	0.172618804709379
**lh temporalpole**	0	0.956595288923278	**rh temporalpole**	0	0.858649119858019
**lh transversetemporal**	0	0.839004785465465	**rh transversetemporal**	0	0.550550833912705
**lh insula**	0	0.790021116339956	**rh insula**	0	0.630572688865819

Notes: Significant p-values of structures indicated in bold. We adjust for sex, age, age^2, sex-by-age, sex-by-age^2, body mass index, ancestry, metabolic/lifestyle variables, higher education, site, and Euler numbers. *Abbreviations:* lh – left hemisphere; r - partial correlation coefficient; rh – right hemisphere.

Supplementary Table 6: Brain volume alterations in probable sarcopenia relative to non-sarcopenia.

**Left hemisphere**			**Right hemisphere**		
**Structure**	**r**	**p-value**	**Structure**	**r**	**p-value**
**L cerebellum cortex**	-0.03	**2.4535370786546e-10**	**R cerebellum cortex**	-0.04	**1.15777646179759e-13**
**L cerebellum white matter**	-0.04	**2.69661774523419e-15**	**R cerebellum white matter**	-0.04	**2.5588212578874e-12**
**L Lateral Ventricle**	0.03	**1.02505050895845e-09**	**R Lateral Ventricle**	0.03	**6.85470831681395e-10**
**L Thalamus Proper**	-0.03	**2.41427514734542e-06**	**R Thalamus Proper**	-0.03	**1.53001909573098e-10**
**L Hippocampus**	-0.02	**4.45020084925974e-05**	**R Hippocampus**	-0.02	**6.06280112753053e-06**
**L Amygdala**	-0.02	0.00312742554849201	**R Amygdala**	-0.02	0.00143594939070124
**L Accumbens area**	-0.01	0.0254244277959708	**R Accumbens area**	-0.03	**3.17404373024634e-06**
**L Caudate**	-0.01	0.0507042447733482	**R Caudate**	-0.01	0.075902778736931
**L Putamen**	-0.01	0.00948101651207635	**R Putamen**	-0.02	**6.23731859041406e-05**
**L Pallidum**	0	0.958581773830082	**R Pallidum**	-0.01	0.0216325363822951
**L Ventral DC**	-0.03	**7.84401108265337e-07**	**R Ventral DC**	-0.04	**1.27952204851909e-13**
**Bilateral structures**					
**brainstem**	-0.05	**2.30073325932995e-23**			
**CSF**	0.02	0.000426549165051966			
**3rd ventricle**	0.02	0.000461901854589412			
**4th ventricle**	-0.02	0.0014393695668852			
**CC posterior**	-0.02	**0.000160671944720132**			
**CC mid-posterior**	-0.03	**1.07071687804349e-08**			
**CC central**	-0.03	**2.9801954696194e-08**			
**CC mid-anterior**	-0.03	**2.69210369966743e-06**			
**CC anterior**	-0.01	0.157268820055934			

Notes: Significant p-values of structures indicated in bold. We adjust for sex, age, age^2, sex-by-age, sex-by-age^2, body mass index, ancestry, metabolic/lifestyle variables, higher education, site, ICV, and Euler numbers. *Abbreviations*: CC - corpus callosum; DC – diencephalon; r - partial correlation coefficient; L – left; R – right.

Supplementary Table 7: White matter microstructure (fractional anisotropy) alterations in probable sarcopenia relative to non-sarcopenia.

**Left hemisphere**			**Right hemisphere**		
**Structure**	**r**	**p-value**	**Structure**	**r**	**p-value**
**CST L**	-0.02	**6.56392599594361e-05**	**CST R**	-0.04	**1.80263498287242e-11**
**ALIC L**	-0.03	**7.01829856340804e-09**	**ALIC R**	-0.04	**5.09252458716411e-12**
**PLIC L**	-0.01	0.0121311365974114	**PLIC R**	-0.03	**2.26283212998877e-10**
**RLIC L**	-0.02	0.00378178319080661	**RLIC R**	-0.02	**2.77273423660998e-05**
**ACR L**	-0.03	**2.23047871627972e-09**	**ACR R**	-0.03	**3.79241373199046e-08**
**SCR L**	-0.02	0.00131230899206003	**SCR R**	-0.02	**3.92098860230034e-05**
**PCR L**	-0.03	**3.45209620174148e-06**	**PCR R**	-0.02	0.00499043516901544
**PTR L**	-0.03	**6.12753483497175e-07**	**PTR R**	-0.02	**6.1209565916109e-06**
**SS L**	-0.03	**3.20090703634619e-07**	**SS R**	-0.03	**3.3812068772184e-06**
**EC L**	-0.03	**3.33298836627389e-08**	**EC R**	-0.03	**7.34177438505664e-08**
**CGC L**	-0.03	**7.23597851382764e-09**	**CGC R**	-0.03	**8.30711974073695e-09**
**CGH L**	-0.02	**4.81928182744591e-05**	**CGH R**	-0.02	0.000635512656191532
**SLF L**	-0.02	0.000265214556580837	**SLF R**	-0.02	0.00213979529004256
**SFO L**	-0.01	0.0140138546675645	**SFO R**	-0.02	0.000496056366687941
**UNC L**	-0.02	**4.90775960572616e-05**	**UNC R**	-0.02	0.000844872323208376
**ICP L**	-0.04	**1.08551266986179e-14**	**ICP R**	-0.04	**2.00223448727501e-12**
**SCP L**	-0.05	**2.14282441591265e-18**	**SCP R**	-0.05	**8.54497808930929e-20**
**CP L**	-0.05	**3.79941780626878e-22**	**CP R**	-0.06	**4.21156090830109e-29**
**ML L**	-0.05	**2.52414406177066e-22**	**ML R**	-0.06	**3.50196730789697e-24**
**TAP L**	-0.02	**3.33235533365332e-05**	**TAP R**	-0.01	0.0188630370432494
**FXST L**	-0.04	**1.0998242989575e-10**	**FXST R**	-0.04	**5.93622349216539e-12**
**Bilateral structures**					
**GCC**	-0.04	**3.89005023991905e-15**			
**BCC**	-0.04	**1.49805132236104e-16**			
**SCC**	-0.04	**3.12898608279277e-13**			
**MCP**	-0.03	**2.61226881430682e-10**			
**P**	-0.02	**5.82857689704685e-05**			
**FX**	-0.04	**8.05401039802666e-13**			

Notes: Significant p-values of structures indicated in bold. We adjust for sex, age, age^2, sex-by-age, sex-by-age^2, body mass index, ancestry, metabolic/lifestyle variables, higher education, and site. *Abbreviations:* L – left; R – Right; r - partial correlation coefficient; *Brainstem tracts*: CST -Corticospinal tract; ML – Medial lemniscus; P – Pontine; MCP – Middle cerebellar peduncle; ICP – Inferior cerebellar peduncle; SCP – Superior cerebellar peduncle. *Projection pathways*: CP – Cerebral peduncle; ACR – Anterior corona radiata; PCR – Posterior corona radiata; SCR – Superior corona radiata; ALIC – Anterior limb of the internal capsule; PLIC – Posterior limb of the internal capsule; RLIC – retrolenticular part of the internal capsule; PTR – Posterior thalamic radiation. *Commissural pathways*: GCC – Genu of corpus callosum; BCC – Body of corpus callosum; SCC – Splenium of the corpus callosum; TAP – Tapetum. *Association pathways:* FX – Fornix; FXST – Fornix stria terminalis; CGC – Cingulum cingulate gyrus; CGH – Cingulum (hippocampal portion); EC – External capsule; SFO – Superior fronto-occipital fasciculus; SLF – Superior longitudinal fasciculus; SS – Sagittal stratum; UNC – Uncinate fasciculus.

Supplementary Table 8: Cortical thickness alterations in probable sarcopenia relative to non-sarcopenia after removing participants with confirmed sarcopenia.

**Left hemisphere**			**Right hemisphere**		
**Structure**	**r**	**p-value**	**Structure**	**r**	**p-value**
**lh bankssts**	-0.01	0.00665171433113433	**rh bankssts**	-0.02	**0.000117823656333449**
**lh caudalanteriorcingulate**	0.01	0.132855308880124	**rh caudalanteriorcingulate**	0.01	0.0410074454654651
**lh caudalmiddlefrontal**	-0.01	0.0153777809538203	**rh caudalmiddlefrontal**	-0.01	0.0974643765456131
**lh cuneus**	-0.01	0.02654553295439	**rh cuneus**	-0.01	0.00651856153704716
**lh entorhinal**	-0.01	0.348552495756547	**rh entorhinal**	-0.01	0.15891222766249
**lh fusiform**	-0.02	0.000115432339582515	**rh fusiform**	-0.02	0.000514045397467373
**lh inferiorparietal**	-0.02	**1.59714763203069e-05**	**rh inferiorparietal**	-0.02	**3.09569863873894e-05**
**lh inferiortemporal**	0	0.74383701181645	**rh inferiortemporal**	0	0.661496241089854
**lh isthmuscingulate**	0.01	0.0547182754120881	**rh isthmuscingulate**	0.01	0.0386563343608998
**lh lateraloccipital**	-0.02	0.00287826376176445	**rh lateraloccipital**	-0.02	0.00529373523901334
**lh lateralorbitofrontal**	0.01	0.0129977380809711	**rh lateralorbitofrontal**	0.01	0.0133014757439318
**lh lingual**	-0.03	**6.17374588070847e-07**	**rh lingual**	-0.02	0.000288645027909011
**lh medialorbitofrontal**	0.01	0.0288238854747851	**rh medialorbitofrontal**	0.01	0.0247854770872721
**lh middletemporal**	-0.01	0.0246274991954218	**rh middletemporal**	-0.03	**2.72859305122785e-07**
**lh parahippocampal**	-0.01	0.0732335150708081	**rh parahippocampal**	-0.01	0.183555320202711
**lh paracentral**	-0.01	0.0120952846821094	**rh paracentral**	-0.01	0.0963293137879959
**lh parsopercularis**	-0.02	**6.65126275867146e-06**	**rh parsopercularis**	-0.02	**0.000168991699713002**
**lh parsorbitalis**	-0.01	0.147902162249771	**rh parsorbitalis**	0	0.599390108524528
**lh parstriangularis**	-0.02	0.00137864304496683	**rh parstriangularis**	-0.02	0.00121594581767198
**lh pericalcarine**	-0.03	**1.47241720480272e-06**	**rh pericalcarine**	-0.03	**5.8490715995523e-07**
**lh postcentral**	-0.03	**1.42116634804728e-09**	**rh postcentral**	-0.03	**2.03558339968879e-09**
**lh posteriorcingulate**	0.01	0.278650701736263	**rh posteriorcingulate**	0.02	0.00379081045724271
**lh precentral**	-0.01	0.00905901921228488	**rh precentral**	-0.02	0.000262784509237137
**lh precuneus**	-0.03	**6.12085477589931e-07**	**rh precuneus**	-0.03	**5.09891992669074e-07**
**lh rostralanteriorcingulate**	0.01	0.0182913829077026	**rh rostralanteriorcingulate**	0.01	0.0275159522455749
**lh rostralmiddlefrontal**	-0.01	0.00734055629133434	**rh rostralmiddlefrontal**	-0.01	0.148437780588944
**lh superiorfrontal**	-0.01	0.107405352390869	**rh superiorfrontal**	0	0.87370466182348
**lh superiorparietal**	-0.02	0.000249180839809837	**rh superiorparietal**	-0.02	**0.000185725405604354**
**lh superiortemporal**	-0.03	**3.17625097457037e-08**	**rh superiortemporal**	-0.03	**3.48738551198598e-08**
**lh supramarginal**	-0.02	0.000317475196322802	**rh supramarginal**	-0.02	0.000438013418481589
**lh frontalpole**	0	0.528494395844959	**rh frontalpole**	0.01	0.0807463085635531
**lh temporalpole**	-0.01	0.0981972982226721	**rh temporalpole**	-0.01	0.0295532528129006
**lh transversetemporal**	-0.02	**5.37340603007284e-06**	**rh transversetemporal**	-0.02	0.00237419049187757
**lh insula**	-0.01	0.180898407568338	**rh insula**	0	0.826964845787595

Notes: Significant p-values of structures indicated in bold. We adjust for sex, age, age^2, sex-by-age, sex-by-age^2, body mass index, ancestry, metabolic/lifestyle variables, higher education, site, and Euler numbers. *Abbreviations:* lh – left hemisphere; r - partial correlation coefficient; rh – right hemisphere.

Supplementary Table 9: Cortical area alterations in probable sarcopenia relative to non-sarcopenia after removing participants with confirmed sarcopenia.

**Left hemisphere**			Right hemisphere		
Structure	**r**	**p-value**	**Structure**	**r**	**p-value**
**lh bankssts**	-0.02	0.00433529549749039	**rh bankssts**	-0.01	0.154610233437557
**lh caudalanteriorcingulate**	0	0.830621590510104	**rh caudalanteriorcingulate**	0	0.816190279109662
**lh caudalmiddlefrontal**	0.01	0.0223299330602354	**rh caudalmiddlefrontal**	0.01	0.0275252451299076
**lh cuneus**	0	0.603824421243029	**rh cuneus**	0	0.828193566907161
**lh entorhinal**	-0.02	0.000505490526219237	**rh entorhinal**	-0.02	0.00211373837213305
**lh fusiform**	-0.02	0.000447066941023089	**rh fusiform**	-0.02	0.00241341421620243
**lh inferiorparietal**	-0.02	0.000534719690472063	**rh inferiorparietal**	-0.01	0.0187612759341382
**lh inferiortemporal**	-0.02	0.00342268045909198	**rh inferiortemporal**	-0.02	0.00236183787719752
**lh isthmuscingulate**	0.01	0.199631242547091	**rh isthmuscingulate**	0	0.530586845906782
**lh lateraloccipital**	0.01	0.268099765756595	**rh lateraloccipital**	0	0.859448707495818
**lh lateralorbitofrontal**	-0.02	0.00198632447586207	**rh lateralorbitofrontal**	-0.01	0.109983489451917
**lh lingual**	-0.01	0.0238750662754243	**rh lingual**	-0.01	0.090694056088489
**lh medialorbitofrontal**	-0.01	0.077381226397984	**rh medialorbitofrontal**	-0.01	0.235915187664757
**lh middletemporal**	-0.02	0.00185536754867961	**rh middletemporal**	-0.02	0.00203264600242218
**lh parahippocampal**	-0.01	0.243259153401906	**rh parahippocampal**	0	0.477633071663986
**lh paracentral**	0.02	0.000320346441615227	**rh paracentral**	0.01	0.0227952749015719
**lh parsopercularis**	0	0.716447357945057	**rh parsopercularis**	0	0.395833207139503
**lh parsorbitalis**	-0.01	0.116190125019601	**rh parsorbitalis**	0	0.380715910005097
**lh parstriangularis**	0	0.965095906594231	**rh parstriangularis**	0	0.935160499945626
**lh pericalcarine**	-0.01	0.0619769010325558	**rh pericalcarine**	-0.01	0.0300494243646498
**lh postcentral**	0	0.845324521080326	**rh postcentral**	0	0.793049444212741
**lh posteriorcingulate**	0	0.628626033766048	**rh posteriorcingulate**	0	0.812627997952147
**lh precentral**	0	0.440530321691706	**rh precentral**	0.01	0.250500753829481
**lh precuneus**	0	0.763714047018835	**rh precuneus**	0	0.681005847468033
**lh rostralanteriorcingulate**	0	0.804325506286424	**rh rostralanteriorcingulate**	0	0.572288371225746
**lh rostralmiddlefrontal**	0.01	0.0423351435376502	**rh rostralmiddlefrontal**	0.01	0.0943263069589606
**lh superiorfrontal**	0.01	0.25430711654284	**rh superiorfrontal**	0	0.371327586779425
**lh superiorparietal**	0	0.7698974557716072	**rh superiorparietal**	0	0.623890322993395
**lh superiortemporal**	0	0.728246776882256	**rh superiortemporal**	0	0.830817698501221
**lh supramarginal**	-0.01	0.275854013255769	**rh supramarginal**	-0.01	0.0986508438010454
**lh frontalpole**	0	0.434459128933782	**rh frontalpole**	0.01	0.22044601778037
**lh temporalpole**	0	0.933112284783634	**rh temporalpole**	0	0.9414240298007
**lh transversetemporal**	0	0.863877322842498	**rh transversetemporal**	0	0.370419948321948
**lh insula**	0	0.93675442859779	**rh insula**	0	0.708596668228328

Notes: Significant p-values of structures indicated in bold. We adjust for sex, age, age^2, sex-by-age, sex-by-age^2, body mass index, ancestry, metabolic/lifestyle variables, higher education, site, and Euler numbers. *Abbreviations:* lh – left hemisphere; r - partial correlation coefficient; rh – right hemisphere.

Supplementary Table 10: Brain volume alterations in probable sarcopenia relative to non-sarcopenia after removing participants with confirmed sarcopenia.

Left hemisphere			Right hemisphere		
Structure	r	p-value	Structure	r	p-value
L cerebellum cortex	-0.03	**3.03811235910997e-09**	R cerebellum cortex	-0.04	**6.37896784651048e-12**
L cerebellum white matter	-0.04	**4.86314773649978e-14**	R cerebellum white matter	-0.04	**4.52849452023414e-11**
L Lateral Ventricle	0.03	**7.10430502901855e-07**	R Lateral Ventricle	0.03	**3.6245924021554e-07**
L Thalamus Proper	-0.02	**7.63549254697787e-06**	R Thalamus Proper	-0.03	**1.41359874069843e-08**
L Hippocampus	-0.02	0.000779271236028162	R Hippocampus	-0.02	**5.75398087866317e-05**
L Amygdala	-0.01	0.00786026382515578	R Amygdala	-0.02	0.0038474742825773
L Accumbens area	-0.01	0.133241471911162	R Accumbens area	-0.02	**3.57128610950757e-05**
L Caudate	-0.01	0.0302804424131694	R Caudate	-0.01	0.0522108862853496
L Putamen	-0.01	0.0289271535799489	R Putamen	-0.02	0.000574785861903289
L Pallidum	0	0.94234822086967	R Pallidum	-0.01	0.0468541257833631
L Ventral DC	-0.03	**5.66289126178883e-07**	R Ventral DC	-0.04	**1.13684872192272e-12**
Bilateral structures					
brainstem	-0.05	**1.09659408147569e-20**			
CSF	0.01	0.00820674702155867			
3rd ventricle	0.02	0.00371583378474135			
4th ventricle	-0.02	0.00159129548865331			
CC posterior	-0.02	**9.44326551813001e-05**			
CC mid-posterior	-0.03	**8.49596803399378e-07**			
CC central	-0.03	**1.17110290444645e-06**			
CC mid-anterior	-0.02	**6.44576216861684e-06**			
CC anterior	-0.01	0.0955090432074194			

Notes: Significant p-values of structures indicated in bold. We adjust for sex, age, age^2, sex-by-age, sex-by-age^2, body mass index, ancestry, metabolic/lifestyle variables, higher education, site, ICV, and Euler numbers. *Abbreviations*: CC - corpus callosum; DC – diencephalon; r - partial correlation coefficient; L – left; R – right.

Supplementary Table 11: White matter (fractional anisotropy) alterations in probable sarcopenia relative to non-sarcopenia after removing participants with confirmed sarcopenia.

Left hemisphere			Right hemisphere		
**Structure**	**r**	**p-value**	**Structure**	**r**	**p-value**
**CST L**	-0.02	0.000358112505221771	CST R	-0.03	**1.15002508632507e-09**
**ALIC L**	-0.03	**2.09623647482907e-07**	ALIC R	-0.03	**8.18723200449276e-10**
**PLIC L**	-0.01	0.00862401979061267	PLIC R	-0.03	**1.56106234422547e-09**
**RLIC L**	-0.01	0.00986080360832751	RLIC R	-0.02	**4.5765920501846e-05**
**ACR L**	-0.03	**3.09387700462676e-07**	ACR R	-0.02	**6.13626052423502e-06**
**SCR L**	-0.02	0.002372048043565	SCR R	-0.02	0.000263789245069761
**PCR L**	-0.02	**2.07486762911154e-05**	PCR R	-0.02	0.00518136267413282
**PTR L**	-0.02	**1.20509132614793e-05**	PTR R	-0.02	**0.000121183114811582**
**SS L**	-0.02	**2.63542534853946e-05**	SS R	-0.02	**4.97949127063816e-05**
**EC L**	-0.03	**6.58722992942078e-07**	EC R	-0.03	**2.21284763356987e-06**
**CGC L**	-0.03	**5.76275359869534e-07**	CGC R	-0.03	**7.51973409768403e-07**
**CGH L**	-0.02	0.000450149672402984	CGH R	-0.02	0.00308788801342634
**SLF L**	-0.02	0.00084561571875917	SLF R	-0.01	0.0141466871553142
**SFO L**	-0.01	0.00817094858057761	SFO R	-0.02	0.00295475118735097
**UNC L**	-0.02	0.000402050797567424	UNC R	-0.02	0.00284546782020594
**ICP L**	-0.04	**1.17183700407723e-12**	ICP R	-0.04	**8.7199408571509e-11**
**SCP L**	-0.05	**1.37038374230727e-17**	SCP R	-0.05	**8.08976859766474e-18**
**CP L**	-0.05	**6.34560606107341e-20**	CP R	-0.06	**2.21317337502212e-26**
**ML L**	-0.05	**8.6491806725406e-19**	ML R	-0.05	**4.23655493936232e-20**
**TAP L**	-0.02	**1.8880798989788e-05**	TAP R	-0.01	0.00915955499498074
**FXST L**	-0.03	**1.1682004662194e-08**	FXST R	-0.03	**5.99095568116802e-10**
**Bilateral structures**					
**GCC**	-0.04	**2.08430312918642e-13**			
**BCC**	-0.04	**4.9517537253058e-14**			
**SCC**	-0.04	**1.44199130337067e-11**			
**MCP**	-0.03	**4.35498943224897e-08**			
**P**	-0.02	**0.000154309939682431**			
**FX**	-0.03	**8.10780014156371e-10**			

Notes: Significant p-values of structures indicated in bold. We adjust for sex, age, age^2, sex-by-age, sex-by-age^2, body mass index, ancestry, metabolic/lifestyle variables, higher education, and site. *Abbreviations:* L – left; R – Right; r - partial correlation coefficient; *Brainstem tracts*: CST -Corticospinal tract; ML – Medial lemniscus; P – Pontine; MCP – Middle cerebellar peduncle; ICP – Inferior cerebellar peduncle; SCP – Superior cerebellar peduncle. *Projection pathways*: CP – Cerebral peduncle; ACR – Anterior corona radiata; PCR – Posterior corona radiata; SCR – Superior corona radiata; ALIC – Anterior limb of the internal capsule; PLIC – Posterior limb of the internal capsule; RLIC – retrolenticular part of the internal capsule; PTR – Posterior thalamic radiation. *Commissural pathways*: GCC – Genu of corpus callosum; BCC – Body of corpus callosum; SCC – Splenium of the corpus callosum; TAP – Tapetum. *Association pathways:* FX – Fornix; FXST – Fornix stria terminalis; CGC – Cingulum cingulate gyrus; CGH – Cingulum (hippocampal portion); EC – External capsule; SFO – Superior fronto-occipital fasciculus; SLF – Superior longitudinal fasciculus; SS – Sagittal stratum; UNC – Uncinate fasciculus.

Supplementary Table 12: The association between cortical thickness and muscle fat infiltration (MFI).

Left hemisphere			Right hemisphere		
**Structure**	**r**	**p-value**	**Structure**	**r**	**p-value**
**lh bankssts**	-0.04	**2.28138644899056e-11**	**rh bankssts**	-0.04	**3.329059781002e-11**
**lh caudalanteriorcingulate**	-0.01	0.101164188807462	**rh caudalanteriorcingulate**	0	0.850669247645672
**lh caudalmiddlefrontal**	-0.05	**1.02854788436433e-17**	**rh caudalmiddlefrontal**	-0.05	**5.3284718570853e-16**
**lh cuneus**	-0.04	**1.7602140852041e-14**	**rh cuneus**	-0.04	**3.09287781719688e-13**
**lh entorhinal**	-0.02	**0.00170017892032311**	**rh entorhinal**	-0.02	0.00754265001007235
**lh fusiform**	-0.04	**1.53504362373672e-11**	**rh fusiform**	-0.04	**3.10024344887734e-12**
**lh inferiorparietal**	-0.05	**1.84641147221558e-17**	**rh inferiorparietal**	-0.05	**1.74296282212058e-16**
**lh inferiortemporal**	-0.01	0.0159991188786958	**rh inferiortemporal**	-0.02	0.000692264733735997
**lh isthmuscingulate**	-0.02	**1.76498073726492e-05**	**rh isthmuscingulate**	-0.02	0.000261902494612845
**lh lateraloccipital**	-0.03	**3.89797549314641e-08**	**rh lateraloccipital**	-0.04	**2.94970906595567e-10**
**lh lateralorbitofrontal**	-0.01	0.141608612256599	**rh lateralorbitofrontal**	-0.01	0.0243745901657608
**lh lingual**	-0.05	**6.7842891734004e-19**	**rh lingual**	-0.05	**1.31373640481422e-15**
**lh medialorbitofrontal**	-0.03	**8.60428742252315e-09**	**rh medialorbitofrontal**	-0.03	**1.69754672106408e-07**
**lh middletemporal**	-0.05	**3.55530511638137e-20**	**rh middletemporal**	-0.04	**1.25215542757637e-12**
**lh parahippocampal**	-0.02	0.00067725973279262	**rh parahippocampal**	-0.03	**3.08350649659807e-06**
**lh paracentral**	-0.03	**3.63893875242553e-08**	**rh paracentral**	-0.03	**2.27446101805851e-09**
**lh parsopercularis**	-0.05	**5.01646966169464e-17**	**rh parsopercularis**	-0.04	**1.25242507351953e-13**
**lh parsorbitalis**	-0.02	**4.25642738319775e-05**	**rh parsorbitalis**	-0.02	0.00036832264366 23
**lh parstriangularis**	-0.04	**1.11190034707594e-13**	**rh parstriangularis**	-0.03	**1.36937332859155e-08**
**lh pericalcarine**	-0.05	**6.37357151907733e-20**	**rh pericalcarine**	-0.04	**8.10125177304955e-14**
**lh postcentral**	-0.06	**6.17259679672149e-24**	**rh postcentral**	-0.06	**1.80585735593499e-26**
**lh posteriorcingulate**	-0.04	**4.89685514338835e-11**	**rh posteriorcingulate**	-0.03	**1.64249539912242e-07**
**lh precentral**	-0.06	**4.29487785912353e-25**	**rh precentral**	-0.05	**9.05234086305378e-21**
**lh precuneus**	-0.06	**1.37822782612778e-23**	**rh precuneus**	-0.06	**1.90305650163104e-26**
**lh rostralanteriorcingulate**	-0.03	**2.48043986220571e-09**	**rh rostralanteriorcingulate**	-0.01	0.052267538574774
**lh rostralmiddlefrontal**	-0.04	**4.72408040095532e-12**	**rh rostralmiddlefrontal**	-0.04	**2.15967585477553e-10**
**lh superiorfrontal**	-0.06	**7.18585478405942e-24**	**rh superiorfrontal**	-0.06	**3.40000437941876e-23**
**lh superiorparietal**	-0.06	**1.5149511102929e-22**	**rh superiorparietal**	-0.05	**1.47452528581656e-20**
**lh superiortemporal**	-0.06	**2.09940589070459e-28**	**rh superiortemporal**	-0.06	**6.58094682038511e-25**
**lh supramarginal**	-0.07	**1.89809212161998e-31**	**rh supramarginal**	-0.06	**3.33368217223657e-23**
**lh frontalpole**	-0.01	0.0246678768629758	**rh frontalpole**	0	0.827427407599355
**lh temporalpole**	-0.02	0.00559140445314839	**rh temporalpole**	-0.01	0.064858306717947
**lh transversetemporal**	-0.05	**2.04575926546772e-15**	**rh transversetemporal**	-0.04	**5.68195361997733e-13**
**lh insula**	-0.05	**1.65680534291022e-16**	**rh insula**	-0.04	**6.66151421612412e-15**

Notes: Significant p-values of structures indicated in bold. We adjust for sex, age, age^2, sex-by-age, sex-by-age^2, body mass index, ancestry, metabolic/lifestyle variables, higher education, site, and Euler numbers. *Abbreviations:* lh – left hemisphere; r - partial correlation coefficient; rh – right hemisphere.

Supplementary Table 13: The association between cortical area and muscle fat infiltration (MFI).

**Left hemisphere**			**Right hemisphere**		
**Structure**	**r**	**p-value**	**Structure**	**r**	**p-value**
**lh bankssts**	0	0.899620777631476	**rh bankssts**	0.01	0.0837240442466076
**lh caudalanteriorcingulate**	0.02	0.000903600724438259	**rh caudalanteriorcingulate**	0.01	0.0317938221754021
**lh caudalmiddlefrontal**	0.02	**6.02274275673072e-05**	**rh caudalmiddlefrontal**	0.03	**1.19370189633668e-05**
**lh cuneus**	-0.02	0.0016522431480955	**rh cuneus**	-0.01	0.0264212942230952
**lh entorhinal**	0	0.623969850962962	**rh entorhinal**	0.01	0.104178212905931
**lh fusiform**	0	0.887683595820502	**rh fusiform**	0	0.687535948672226
**lh inferiorparietal**	-0.01	0.254954760974025	**rh inferiorparietal**	0	0.450523669245103
**lh inferiortemporal**	0	0.74398410685634	**rh inferiortemporal**	0	0.539112101609918
**lh isthmuscingulate**	0.03	**4.36643177599069e-07**	**rh isthmuscingulate**	0.02	0.00162737847974414
**lh lateraloccipital**	0.01	0.260203741179018	**rh lateraloccipital**	0	0.476360925624352
**lh lateralorbitofrontal**	0.01	0.0366379024494297	**rh lateralorbitofrontal**	0.01	0.0635526712430553
**lh lingual**	-0.01	0.312919850048539	**rh lingual**	0.01	0.363864013718812
**lh medialorbitofrontal**	0.01	0.0162460913062854	**rh medialorbitofrontal**	0.02	0.000372651272033442
**lh middletemporal**	-0.01	0.0161297585791522	**rh middletemporal**	0	0.853875261530665
**lh parahippocampal**	0	0.46353428899504	**rh parahippocampal**	0.01	0.105592970690979
**lh paracentral**	0.03	**5.59472385769545e-06**	**rh paracentral**	0.02	0.00034930453984775
**lh parsopercularis**	0.01	0.258723011160621	**rh parsopercularis**	0.01	0.207930697001544
**lh parsorbitalis**	0	0.699382841414328	**rh parsorbitalis**	0	0.991980656955469
**lh parstriangularis**	-0.01	0.0214042003946227	**rh parstriangularis**	-0.01	0.309041375791122
**lh pericalcarine**	-0.02	0.00208885397043273	**rh pericalcarine**	-0.02	0.00145872311927214
**lh postcentral**	0	0.516426436078771	**rh postcentral**	0	0.497028861960248
**lh posteriorcingulate**	0.01	0.0216911665774645	**rh posteriorcingulate**	0.02	**0.000113527980978347**
**lh precentral**	0	0.762579461372567	**rh precentral**	0	0.71600202940458
**lh precuneus**	0.02	0.000628536550900606	**rh precuneus**	0.02	0.000391207642612611
**lh rostralanteriorcingulate**	0.04	**3.21177950728731e-10**	**rh rostralanteriorcingulate**	0.02	0.00309990508338624
**lh rostralmiddlefrontal**	0.01	0.0942031446241146	**rh rostralmiddlefrontal**	0.01	0.191992703902448
**lh superiorfrontal**	0.02	0.000339935015847927	**rh superiorfrontal**	0.02	0.0015868049153481
**lh superiorparietal**	0.01	0.045142879977467	**rh superiorparietal**	0.01	0.0106601410774301
**lh superiortemporal**	0.01	0.0301776375704009	**rh superiortemporal**	0.02	0.00146081219960203
**lh supramarginal**	0	0.695357681167804	**rh supramarginal**	0	0.766992907750803
**lh frontalpole**	0	0.689817683929387	**rh frontalpole**	0	0.437228090388853
**lh temporalpole**	0	0.406622824931882	**rh temporalpole**	0	0.54194959916823
**lh transversetemporal**	0.02	0.00156020809737273	**rh transversetemporal**	0	0.400671821178121
**lh insula**	0.02	0.00259158480399647	**rh insula**	0.02	0.00376540591820498

Notes: Significant p-values of structures indicated in bold. We adjust for sex, age, age^2, sex-by-age, sex-by-age^2, body mass index, ancestry, metabolic/lifestyle variables, higher education, site, and Euler numbers. *Abbreviations:* lh – left hemisphere; r - partial correlation coefficient; rh – right hemisphere.

Supplementary Table 14: The association between brain volumes and muscle fat infiltration (MFI).

**Left hemisphere**			**Right hemisphere**		
**Structure**	**r**	**p-value**	**Structure**	**r**	**p-value**
**L cerebellum cortex**	-0.03	**1.88968831530262e-08**	**R cerebellum cortex**	-0.02	**8.47512098971238e-05**
**L cerebellum white matter**	0.01	0.127346673767964	**R cerebellum white matter**	0	0.532842865547498
**L Lateral Ventricle**	0.04	**9.53139198940093e-12**	**R Lateral Ventricle**	0.04	**3.93849895281661e-12**
**L Thalamus Proper**	0.01	0.265182117613988	**R Thalamus Proper**	-0.03	**3.34766039146789e-06**
**L Hippocampus**	-0.02	0.000306962073557681	**R Hippocampus**	-0.02	**2.07787684151016e-05**
**L Amygdala**	-0.01	0.352878593832847	**R Amygdala**	0.01	0.207217431391738
**L Accumbens-area**	0.01	0.0317056278846247	**R Accumbens area**	0	0.828915573301139
**L Caudate**	0	0.821975395808113	**R Caudate**	0	0.958256521637423
**L Putamen**	-0.01	0.232089194226306	**R Putamen**	-0.01	0.366865140967884
**L Pallidum**	-0.01	0.0264542130990135	**R Pallidum**	-0.01	0.0776518214044681
**L Ventral DC**	0	0.53327457621767	**R Ventral DC**	-0.02	0.00144472396376645
**Bilateral structures**					
**brainstem**	-0.03	**8.6815522789464e-07**			
**CSF**	0.04	**4.51107886479651e-13**			
**3rd ventricle**	0.04	**3.09242675181065e-12**			
**4th ventricle**	0.01	0.0157879153119321			
**CC posterior**	-0.02	0.000670291034526266			
**CC mid-posterior**	-0.04	**3.86707851316786e-10**			
**CC central**	-0.03	**5.97439758595438e-06**			
**CC mid-anterior**	-0.02	0.000706073307858449			
**CC anterior**	-0.02	0.000722846185425296			

Notes: Significant p-values of structures indicated in bold. We adjust for sex, age, age^2, sex-by-age, sex-by-age^2, body mass index, ancestry, metabolic/lifestyle variables, higher education, site, ICV, and Euler numbers. *Abbreviations*: CC - corpus callosum; DC – diencephalon; r - partial correlation coefficient; L – left; R – right.

Supplementary Table 15: The association between white matter microstructure (fractional anisotropy) and muscle fat infiltration (MFI).

**Left hemisphere**			**Right hemisphere**		
**Structure**	**r**	**p-value**	**Structure**	**r**	**p-value**
**CST L**	0	0.427814884351496	**CST R**	-0.01	0.0925495729506713
**ALIC L**	-0.04	**2.01099867648303e-12**	**ALIC R**	-0.03	**1.30181255914633e-09**
**PLIC L**	-0.03	**8.83355795186153e-07**	**PLIC R**	-0.01	0.131281397201807
**RLIC L**	-0.03	**8.40087494196272e-07**	**RLIC R**	-0.01	0.0111531088799668
**ACR L**	-0.02	**1.47116891732123e-05**	**ACR R**	-0.02	**4.3335519460488e-05**
**SCR L**	-0.01	0.0237879164961693	**SCR R**	-0.01	0.0124513945613348
**PCR L**	-0.01	0.0524039486442859	**PCR R**	0	0.47714494789201
**PTR L**	-0.04	**8.78498931494452e-10**	**PTR R**	-0.03	**1.41670784721672e-07**
**SS L**	-0.04	**1.10260156671674e-12**	**SS R**	-0.03	**4.06476620233617e-07**
**EC L**	-0.03	**1.04178289958825e-05**	**EC R**	-0.03	**2.8934975150558e-06**
**CGC L**	-0.03	**1.65717973567808e-06**	**CGC R**	-0.03	**9.81722855275246e-07**
**CGH L**	-0.02	**3.33640728554539e-05**	**CGH R**	-0.02	0.000333823589084817
**SLF L**	-0.01	0.0298719986965192	**SLF R**	-0.01	0.0301646959099631
**SFO L**	-0.01	0.125350775840129	**SFO R**	-0.02	0.00820112811144784
**UNC L**	-0.01	0.0219634884674666	**UNC R**	-0.01	0.0293937031573015
**ICP L**	-0.03	**5.36249282018476e-07**	**ICP R**	-0.03	**8.01931131872615e-08**
**SCP L**	-0.04	**3.68195497876637e-12**	**SCP R**	-0.04	**1.88517866786725e-12**
**CP L**	-0.04	**6.0822900533276e-13**	**CP R**	-0.04	**4.84319475466224e-11**
**ML L**	-0.03	**4.95864863945222e-06**	**ML R**	-0.02	**2.15089116109993e-05**
**TAP L**	-0.01	0.0842976354925605	**TAP R**	-0.01	0.343991419342909
**FXST L**	-0.04	**1.3100107742608e-11**	**FXST R**	-0.04	**1.49202765425997e-11**
**Bilateral structures**					
**GCC**	-0.05	**1.09372633734309e-17**			
**BCC**	-0.04	**8.64887372700057e-12**			
**SCC**	-0.03	**1.80580480282466e-06**			
**MCP**	-0.02	**0.000174522878930683**			
**P**	0.01	0.337350906373164			
**FX**	-0.04	**7.89000873558208e-12**			

Notes: Significant p-values of structures indicated in bold. We adjust for sex, age, age^2, sex-by-age, sex-by-age^2, body mass index, ancestry, metabolic/lifestyle variables, higher education, and site. *Abbreviations:* L – left; R – Right; r - partial correlation coefficient; *Brainstem tracts*: CST -Corticospinal tract; ML – Medial lemniscus; P – Pontine; MCP – Middle cerebellar peduncle; ICP – Inferior cerebellar peduncle; SCP – Superior cerebellar peduncle. *Projection pathways*: CP – Cerebral peduncle; ACR – Anterior corona radiata; PCR – Posterior corona radiata; SCR – Superior corona radiata; ALIC – Anterior limb of the internal capsule; PLIC – Posterior limb of the internal capsule; RLIC – retrolenticular part of the internal capsule; PTR – Posterior thalamic radiation. *Commissural pathways*: GCC – Genu of corpus callosum; BCC – Body of corpus callosum; SCC – Splenium of the corpus callosum; TAP – Tapetum. *Association pathways:* FX – Fornix; FXST – Fornix stria terminalis; CGC – Cingulum cingulate gyrus; CGH – Cingulum (hippocampal portion); EC – External capsule; SFO – Superior fronto-occipital fasciculus; SLF – Superior longitudinal fasciculus; SS – Sagittal stratum; UNC – Uncinate fasciculus.

Supplementary Table 16: The results of the mediation analyses for probable sarcopenia relative to non-sarcopenia.

Structure	Effect direction	Estimate	Standard error	z-score	p-value
Superior temporal thickness	**direct**	-0.320523463475367	0.0506834847413481	-6.32402182113339	**2.54841703295483e-10**
	**indirect**	-0.00962426029843068	0.00298695040054232	-3.22210248174301	0.00127253610376465
	**total**	-0.330147723773797	0.0509205717620393	-6.48358241766481	**8.95699070468936e-11**
Postcentral thickness	**direct**	-0.318798175239106	0.0507791810535172	-6.27812754410351	**3.42674777442653e-10**
	**indirect**	-0.0113495485346927	0.00282733786116086	-4.01421729274079	**5.96433823458753e-05**
	**total**	-0.330147723773798	0.0507933862893016	-6.49981715913547	**8.04176725210937e-11**
FA Body of Corpus Callosum (BCC)	**direct**	-0.320749100943182	0.0509119409859181	-6.3000760672609	**2.97499580526051e-10**
	**indirect**	-0.00939862283061028	0.00270709324950827	-3.47185041827336	0.000516884254455174
	**total**	-0.330147723773792	0.0510266727120214	-6.47010095361387	**9.79374359388885e-11**
FA Genu of Corpus Callosum (GCC)	**direct**	-0.314631454949384	0.0504684137637344	-6.2342251615499	**4.54019710716125e-10**
	**indirect**	-0.0155162688244053	0.0040090590760696	-3.87030186634643	**0.000108700671567652**
	**total**	-0.330147723773789	0.0507049762200385	-6.51115035220775	**7.45774553223555e-11**
FA Medial Lemniscus (ML)	**direct**	-0.306437707241296	0.051582028335233	-5.94078436097451	**2.83661538702518e-09**
	**indirect**	-0.0237100165325026	0.00415387797609359	-5.70792321511574	**1.14362987968519e-08**
	**total**	-0.330147723773799	0.0515633982922921	-6.4027534008198	**1.52599488600913e-10**
FA Cerebral Peduncle (CP)	**direct**	-0.288871204499343	0.0513519809196851	-5.62531764745627	**1.85166921795599e-08**
	**indirect**	-0.0412765192744497	0.00606759599002764	-6.80277977345385	**1.02620134612152e-11**
	**total**	-0.330147723773792	0.051434176017207	-6.41883956036048	**1.37317046622343e-10**
FA Superior Cerebellar Peduncle (SCP)	**direct**	-0.305829031867119	0.0509939423953742	-5.99736002947013	**2.00551086848577e-09**
	**indirect**	-0.0243186919066768	0.00500177251475613	-4.86201478274597	**1.16196942800961e-06**
	**total**	-0.330147723773796	0.0510299577002517	-6.46968444914403	**9.82078862676872e-11**
Brainstem	**direct**	-0.28997823430321	0.0509844342236055	-5.68758364624456	**1.28849495517613e-08**
	**indirect**	-0.0228780326314985	0.00483037340631881	-4.73628655738514	**2.17669757218708e-06**
	**total**	-0.312856266934709	0.050963251892536	-6.13885996903053	**8.31157809244587e-10**
Lateral ventricle	**direct**	-0.330474652610327	0.0514899233897671	-6.41823935352766	**1.3785927954757e-10**
	**indirect**	0.000608935228076904	0.00161067368455102	0.378062443012253	0.705384207442564
	**total**	-0.32986571738225	0.0514807350961705	-6.40755647265006	**1.4787038260522e-10**
Cerebellum cortex	**direct**	-0.308836306969834	0.0510770405664264	-6.04648005336541	**1.48044554393323e-09**
	**indirect**	-0.0106151365363334	0.0044246714072483	-2.39907906357615	0.0164363653382489
	**total**	-0.319451443506167	0.0511346904083531	-6.24725486660975	**4.17729184576388e-10**
Cerebellum white matter (WM)	**direct**	-0.301030260133685	0.050286208196056	-5.98633842026878	**2.14617590366117e-09**
	**indirect**	-0.0184519566657632	0.00402704952051097	-4.582003914226	**4.60541272562232e-06**
	**total**	-0.319482216799448	0.0503384872825105	-6.34667893388302	**2.20012674745362e-10**

Notes: Significant p-values of direct, indirect, and total effects are indicated in bold. We adjust for sex, age, age^2 body mass index, ancestry, metabolic/lifestyle variables, higher education, site, ICV (except FA and cortical thickness), and Euler numbers (T1-weighted MRI). *Abbreviations:* FA – fractional anisotropy.

Supplementary Table 17: The results of the mediation analyses for muscle fat infiltration (MFI).

Structure	Effect direction	Estimate	Standard error	z-score	p-value
Superior temporal thickness	**direct**	-0.0662342247671658	0.00745746020266028	-8.88160619932484	**0**
	**indirect**	-0.00410237105357244	0.000690944437890346	-5.93733855952032	**2.89686052923344e-09**
	**total**	-0.0703365958207382	0.00744543893953778	-9.44693743269148	**0**
Postcentral thickness	**direct**	-0.0677135437438751	0.00744480853799825	-9.09540432077812	**0**
	**indirect**	-0.00262305130539191	0.000575190425501674	-4.56031809483636	**5.10761908589785e-06**
	**total**	-0.070336595049267	0.00742694181468775	-9.47046534149051	**0**
FA Body of Corpus Callosum (BCC)	**direct**	-0.0686735042565763	0.00733133125549513	-9.36712608710768	**0**
	**indirect**	-0.00166309105589208	0.000420723020297573	-3.95293572173872	**7.71981800893329e-05**
	**total**	-0.0703365953124683	0.00733839136265218	-9.58474300927007	**0**
FA Genu of Corpus Callosum (GCC)	**direct**	-0.0672496985308519	0.00735040595623739	-9.14911352260555	**0**
	**indirect**	-0.00308689742945109	0.0005912398371757	-5.22105791145083	**1.77903892284448e-07**
	**total**	-0.070336595960303	0.00736784542577938	-9.54642665469093	**0**
FA Medial Lemniscus (ML**)**	**direct**	-0.0686367364821151	0.00736046254289923	-9.3250575058398	**0**
	**indirect**	-0.00169985847151679	0.000481046042019644	-3.53367104816004	0.000409830807247547
	**total**	-0.0703365949536319	0.00736183227687758	-9.55422404481404	**0**
FA Cerebral Peduncle (CP)	**direct**	-0.0648267920623048	0.00731255919248231	-8.86513057274807	**0**
	**indirect**	-0.00550979397661375	0.000843841709930106	-6.52941649100293	**6.60265175866925e-11**
	**total**	-0.0703365860389186	0.00735669145499989	-9.56089928049316	**0**
FA Superior Cerebellar Peduncle (SCP)	**direct**	-0.0653197764397294	0.00739007799723522	-8.83884804249249	**0**
	**indirect**	-0.00501681940744655	0.000730189368392134	-6.8705730658521	**6.39444053263105e-12**
	**total**	-0.0703365958471759	0.00739722303525969	-9.50851360191638	**0**
Brainstem	**direct**	-0.0601101038361254	0.00736308916817684	-8.16370717007209	**2.22044604925031e-16**
	**indirect**	-0.00516872303837976	0.000727123958956057	-7.10844825660892	**1.17350573702879e-12**
	**total**	-0.0652788268745051	0.00733375050520565	-8.90115184968031	**0**
Lateral ventricle	**direct**	-0.0703882004037309	0.00741525080718086	-9.49235598822465	**0**
	**indirect**	0.000235476232609607	0.000302214835540678	0.779168342905232	0.435880555655992
	**total**	-0.0701527241711213	0.00741380023128819	-9.46245137211256	**0**
Cerebellum cortex	**direct**	-0.0626030121037388	0.007280969852576	-8.59816938832536	**0**
	**indirect**	-0.00458687112516593	0.000701524394151419	-6.53843424891066	**6.21662721300709e-11**
	**total**	-0.0671898832289047	0.00726347880536352	-9.25037231185836	**0**
Cerebellum white matter (WM)	**direct**	-0.0666160658290154	0.00732369657138514	-9.09596201586164	**0**
	**indirect**	- 0.000499803412765629	0.00054681480912919	- 0.914026841302219	0.360702741957653
	**total**	-0.067115869241781	0.00731225128869066	-9.17855070784895	**0**

Notes: Significant p-values of direct, indirect, and total effects are indicated in bold. We adjust for sex, age, age^2 body mass index, ancestry, metabolic/lifestyle variables, higher education, site, ICV (except FA and cortical thickness), and Euler numbers (T1-weighted MRI). *Abbreviations:* FA – fractional anisotropy. *Abbreviations:* FA – fractional anisotropy.

Supplementary Figures

Supplementary Figure 1: Overview of the included cortical parcellations.

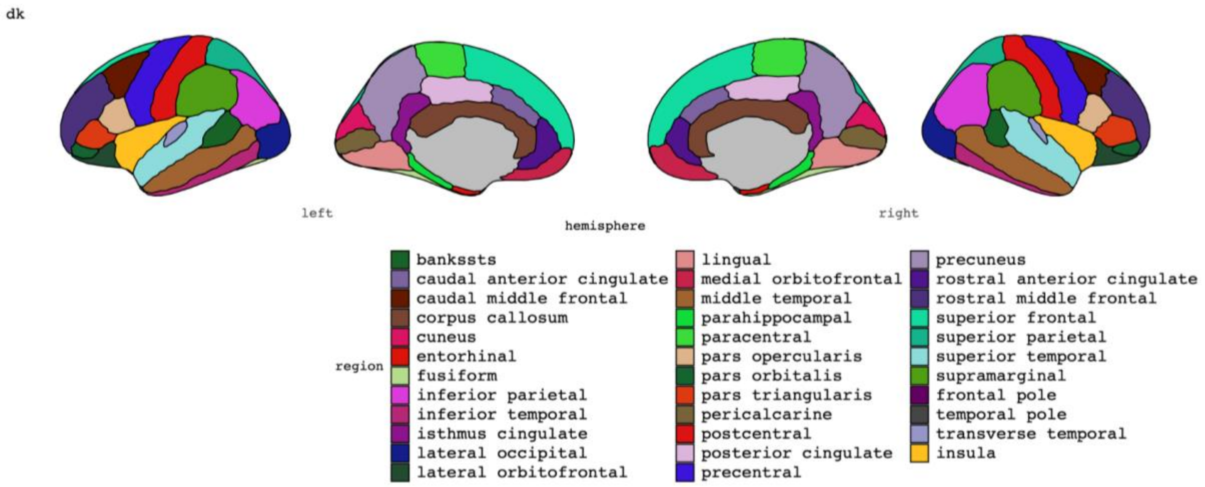

Notes: We used the program ggseg[1] to display cortical parcellations from the FreeSurfer Desikan-Killiany atlas.[2] The colour coding highlights the location of the cortical parcellations included in this work.

Supplementary Figure 2: Overview of the included deep and lower brain structures.

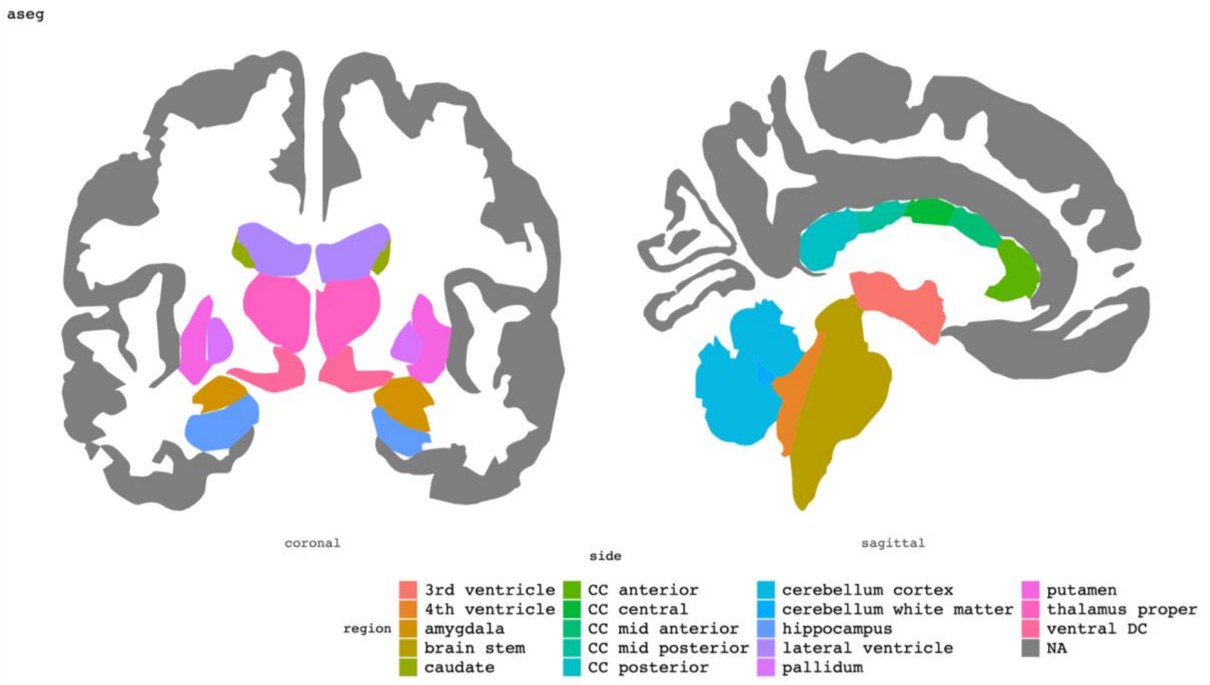

Notes: The color-coding highlights and indicates the location of the various deep and lower brain structures on the aseg atlas, illustrated by *ggseg*.[1] Although not displayed, we also include the accumbens and cerebrospinal fluid (CSF) volumes. *Abbreviations: CC* - corpus callosum; DC – diencephalon.

Supplementary Figure 3: Principal component analysis across cognitive test statistics.

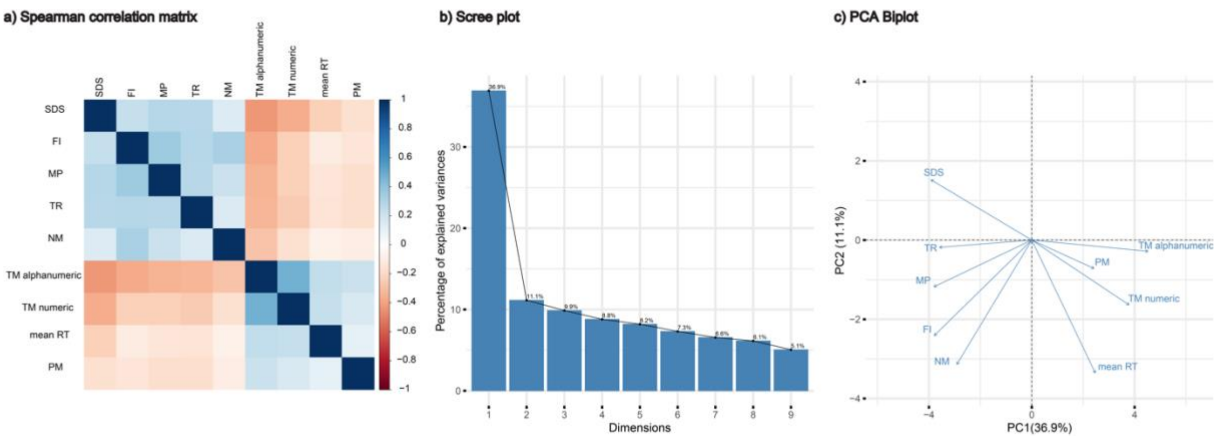

Notes: The figure shows the (a) spearman cross-correlation matrix of the included cognitive tests, (b) PCA scree plot, and (c) PCA biplot. The first principal component (PC1) accounts for 36.9% of the total variance, while the second (PC2) accounts for 11.1%. Five cognitive tests are negatively correlated with PC1: symbol digits substitution (SDS; loading=-0.37), tower rearranging (TR; loading=-0.34), matrix pattern (MP; loading=-0.36), fluid intelligence (FI; loading=-0.36), and numeric memory (NM; loading=-0.28). Four tests are positively correlated with PC1: alphanumeric trail making (TM alphanumeric; loading=0.43), pairs matching (PM; loading=0.23), numeric trail making (TM numeric; loading=0.36), and mean response time (mean RT; loading=0.23). Except for symbol digit substitution, all cognitive tests contribute negatively to PC2.

Supplementary Figure 4: Probable sarcopenia vs. non-sarcopenia on the cortical area.

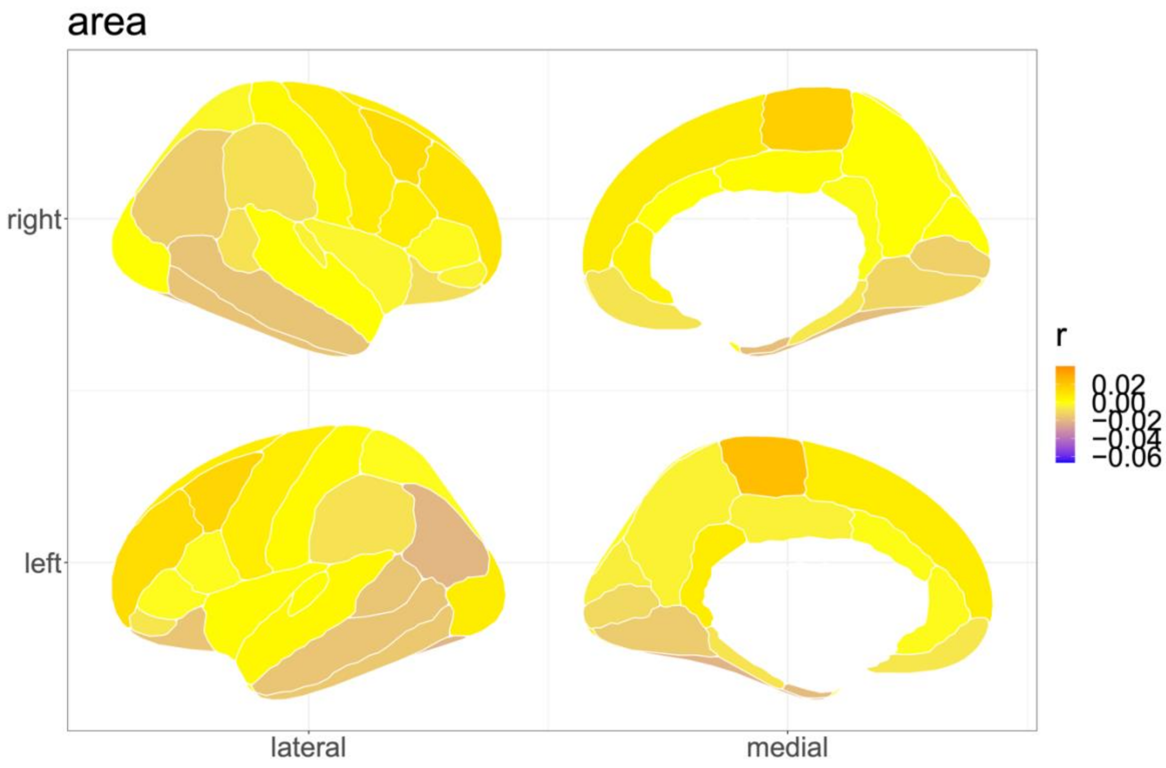

Notes: We used multiple linear regression to obtain the effect-size maps for probable sarcopenia (n=1,704) relative to non-sarcopenia (n=32,171) on the cortical area. We adjust for sex, age, age^2, sex-by-age, sex-by-age^2, BMI, ancestry, metabolic/lifestyle variables, higher education, site, ICV, and Euler numbers. *Abbreviations:* BMI – Body mass index; ICV – intracranial volume.

Supplementary Figure 5: Sensitivity analyses for probable sarcopenia vs. non-sarcopenia after removing the confirmed sarcopenia participants.

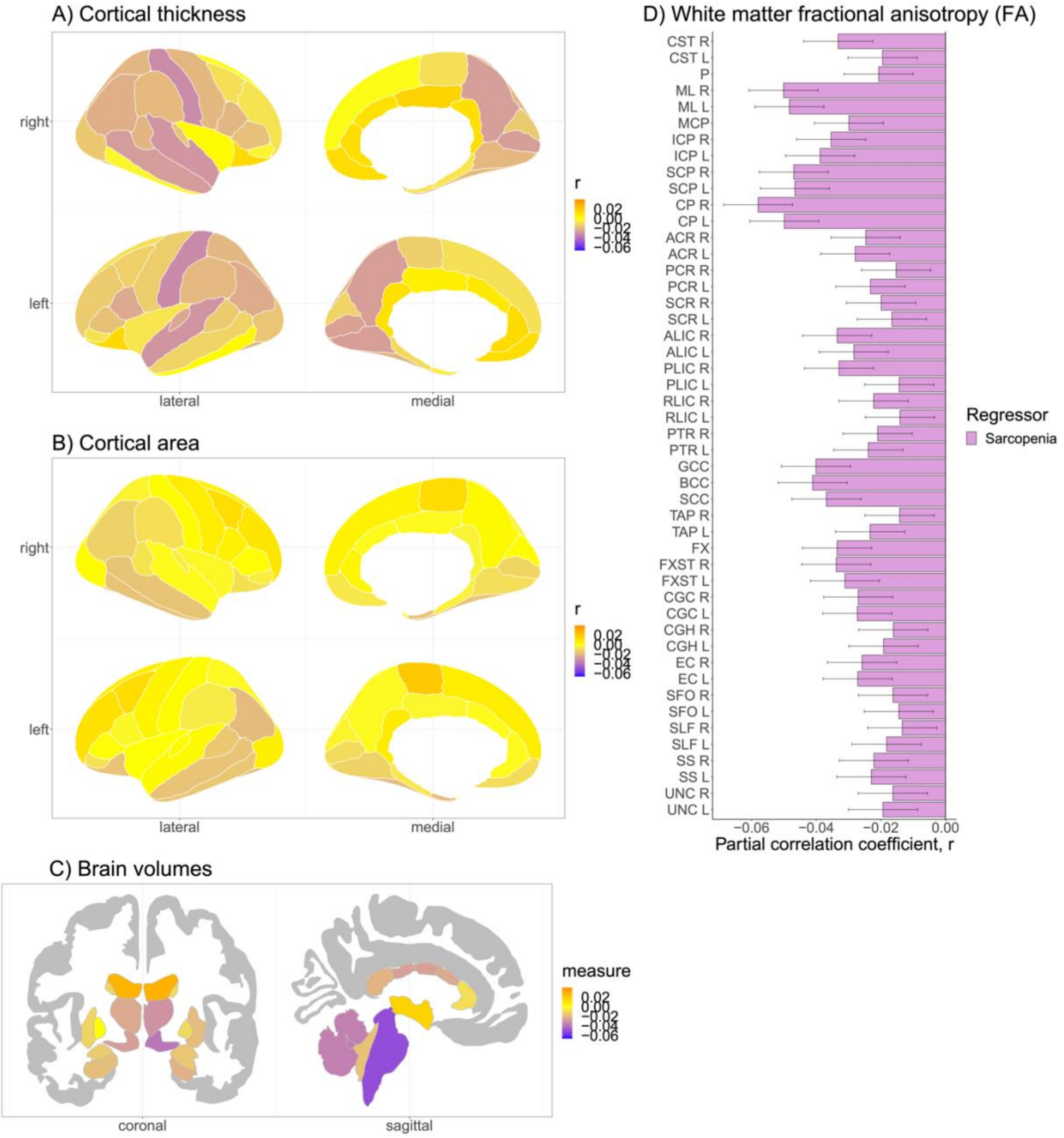

Notes: We used multiple linear regression to obtain the effect-size maps for probable sarcopenia (n=1,541) relative to non-sarcopenia (n=32,171) on (**A**) cortical thickness, (**B**) cortical area, (**C**) brain volumes, and (**D**) white matter fractional anisotropy (FA). For white matter FA, we additionally display the 95% confidence interval. Significant r effect sizes are |r| in [*0.02*, *0.06*] and p-values in [*0.0002*, *2.2e-26*]. We adjust for sex, age, age^2, sex-by-age, sex-by-age^2, BMI,

ancestry, metabolic/lifestyle variables, higher education, site, and ICV and Euler numbers when applicable. *Abbreviations:* BMI – Body mass index; ICV – intracranial volume.

Supplementary Figure 6: Total muscle fat infiltration on the cortical area.

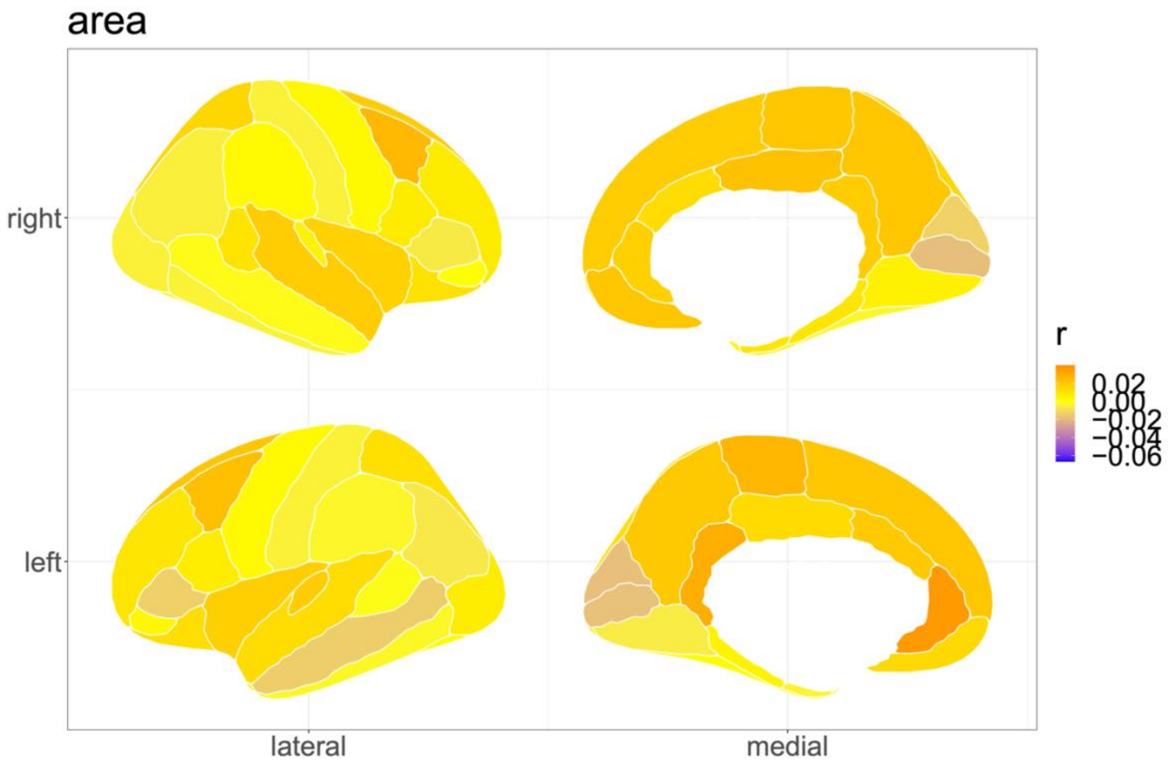

Notes: We used multiple linear regression to obtain the effect-size maps for total muscle fat infiltration (MFI; n=30,561) on the cortical area. We adjust for sex, age, age^2, sex-by-age, sex-by-age^2, BMI, ancestry, metabolic/lifestyle variables, higher education, site, ICV, and Euler numbers. *Abbreviations:* BMI – Body mass index; ICV – intracranial volume.

Supplementary Notes

Supplementary Note 1: Extracted UK Biobank Field-IDs

We extracted the below-outlined Field-IDs from the UK Biobank repository (**Supplementary Tables N1-N2**).

We derived a yes/no variable for having higher education (yes: university or professional degree; no: otherwise). Further, we extracted the maximum handgrip strength (left or right). We computed the fat-free appendicular mass as the sum of the fat-free mass of the arms and legs (kg), used a conversion equation[3] to estimate the appendicular lean mass (ALM; **Supplementary Note 3**), and computed ALM divided by standing height in meter squared (i.e., ALM/height^2).[4] We derived the waist-to-hip ratio (WHR) as waist divided by hip circumference.

Supplementary Table N1: Overview of extracted UK Biobank data Field-IDs

**Field-ID**	**Description**	**Field-ID**	**Description**
31	Sex (from the central registry, but may be updated by the participant)	49	Hip circumference (cm)
54	Assessment centre	46	Handgrip strength (kg; left)
21003	Age (years)	47	Handgrip strength (kg; right)
6138	Education	2296	Falls in the last year (no fall, one fall, more than one fall)
20117	Alcohol drinker status (current, previous, never)	924	Usual walking pace (slow, average, brisk)
20116	Smoking status (current, previous, never)	864	Number of days walking 10+ minutes (field records those unable to walk)
20002	Self-reported illness (non-cancer)	874	Duration of walks (on a typical day; minutes/day)
21000	Self-reported ethnic background	23125	Arm fat-free mass (left; kg)
50	Standing height (cm)	23121	Arm fat-free mass (right; kg)
21002	Weight (kg)	23117	Leg fat-free mass (left; kg)
21001	BMI (kg/m^2)	23113	Leg fat-free mass (right; kg)
48	Waist circumference (cm)	914	Duration of vigorous activity (minutes/typical day)
		894	Duration of moderate activity (minutes/typical day)

Notes: We extracted variables from the imaging time-point when available. For self-reported ethnic background, we complemented missing data with data from the baseline assessment.

Supplementary Table N2: The Included cognitive tests.

Field-IDs	***Cognitive test***	***Scores used***
20023	Mean reaction Time	Mean time in milliseconds (ms) to correctly identify matches and is a rough measure of the processing & reaction speed.
20016	Fluid Intelligence score	Yields the number of correct answers when testing capacity to solve problems that require logics and reasoning. Unattempted questions yield a score of 0.
6348, 6350	Trail Making[1]	Time to complete the numeric AND the alphanumeric path.
4282	Numeric memory[2]	Maximum number of digits remembered correctly.
6373	Matrix pattern completion	The number of puzzles solved correctly.
21004	Tower Rearranging	Number of puzzles solved correct.
23324	Symbol Digit Substitution	The number of symbol digits matches correctly.
399, 400	Pairs Matching[3]	The sum the number of incorrect matches across two rounds.

[1] 0 represents «trail not completed» and we coded this as NA.
[2] -1 indicates an abandoned test, and we coded this as NA.
[3] 0 indicates no errors. There were two rounds, and we computed the sum of incorrect matches across the two rounds. We code participants that did not complete the test as NA (Derived from Field-ID 400 where 0 indicates test not completed).

Supplementary Note 2: Details of UK Biobank Assessment

Handgrip Strength

The handgrip strength assessment of both hands was assessed from an upright sitting position using a Jamar J00105 hydraulic hand dynamometer calibrated at the start of each day. The right hand is measured first, then the left. During the assessment, the participant's upper arm is held along their side, with their elbow at a 90° angle, their forearm pointing forwards with their thumb uppermost, and a straight wrist with hands pointing forward or bent slightly outwards. The assessment takes about 3 seconds per hand and yields a measure of handgrip strength in whole kilograms (kg).

Whole-Body Bioimpedance

The whole-body bioimpedance assessment was performed barefoot in a standing position using a Tanita BC 418ma body composition device. Bioimpedance passes a low electrical current through the body and uses the speed of the electrical current to derive body composition measures. It yields various measures, including fat and fat-free mass measures of the whole body, trunk, arms, and legs, and other measures such as body fat percentage (whole-body and regional), weight, and BMI. Bioimpedance is not available for participants unable to stand or grip handles, unwilling to remove their shoes, wearing a plaster cast, or who were pregnant, an amputee, or using a pacemaker.

Cognitive Test Battery

The UK Biobank deployed a cognitive test battery explicitly developed for population-based cognitive testing,[5] reportedly moderate to highly correlated with standard tests.[6] The UK Biobank cognitive assessment and assessment battery is previously described in detail.[5–7] It was performed via fully automated, unsupervised, touchscreen assessment where the participants completed various cognitive tests. In **Supplementary Table N2**, we list the included cognitive tests of this study.

We did not include all available tests due to the following reasons: (i) no access to data at the time of the study (picture vocabulary, paired association learning); (2) test scores differed too

much from the other included test scores (prospective memory result); and (3) cognitive tests that were assessed part of pilot studies (word production, lights pattern memory). We did not include data from the intermediate online cognitive assessments.

Supplementary Note 3: Estimate Appendicular Lean Mass

A conversion equation is needed for appendicular fat-free mass measures since bioimpedance estimates the muscle mass but does not measure muscle mass directly (see the *European Working Group on Sarcopenia in Older People* (EWGSOP)[4] for details).

To estimate the appendicular lean mass (ALM) from bioimpedance appendicular fat-free mass, we used the following conversion equation:

$$\text{ALM (kg)} = (0.958 \times [\text{Appendicular fat-free mass (kg)}]) - 0.166 \times \text{G} - 0.308,$$

where G is 0 for women and 1 for men. For further details, see Dodds et al. (2020).[3]

Supplementary Note 4: Diffusion MRI Processing

We post-process the diffusion-MRI DICOM data using an optimized post-processing pipeline.[8] It includes corrections for noise,[9] Gibs ringing,[10] geometric echo-planar imaging distortion (*FSL topup*),[11,12] eddy-current and motion-induced distortions (*FSL Eddy*),[13–15] and B_0 field inhomogeneity,[16] before applying isotropic 1mm^3 Gaussian kernel smoothing (*FSL fslmaths*). We derived the following diffusion maps: fractional anisotropy (FA), radial diffusivity (RD), axial diffusivity (AD), and mean diffusivity (MD). We applied *tract-based spatial statistics* (TBSS) to extract diffusion metrics.[17] We used nonlinear transformation (*FSL FNIRT*) to align all diffusion maps to the *FSL FMRI58_FA template*.[8] Subsequently, we derived the mean FA map across all participants and thinned it to create the mean FA skeleton before projecting the scalar diffusion maps onto the FA skeleton. Lastly, we extracted the mean over the skeleton (i.e., global diffusion) and 27 regions of interest (ROIs; **Supplementary Table 3**) from the Johns Hopkins University (JHU) labeled DTI atlas.[18]

Supplementary references

1. Mowinckel AM, Vidal-Piñeiro D. Visualization of Brain Statistics With R Packages ggseg and ggseg3d. *Advances in Methods and Practices in Psychological Science*. 2020;3(4):466-483. doi:10.1177/2515245920928009

2. Desikan RS, Segonne F, Fischl B, et al. An automated labeling system for subdividing the human cerebral cortex on MRI scans into gyral based regions of interest. *NeuroImage*. 2006;31(3):968-980. doi:10.1016/j.neuroimage.2006.01.021

3. Dodds RM, Granic A, Robinson SM, Sayer AA. Sarcopenia, long-term conditions, and multimorbidity: findings from UK Biobank participants. *Journal of Cachexia, Sarcopenia and Muscle*. 2020;11(1):62-68. doi:10.1002/jcsm.12503

4. Cruz-Jentoft AJ, Bahat G, Bauer J, et al. Sarcopenia: revised European consensus on definition and diagnosis. *Age and Ageing*. 2019;48(1):16-31. doi:10.1093/ageing/afy169

5. Cornelis MC, Wang Y, Holland T, Agarwal P, Weintraub S, Morris MC. Age and cognitive decline in the UK Biobank. *PLOS ONE*. 2019;14(3):e0213948. doi:10.1371/journal.pone.0213948

6. Fawns-Ritchie C, Deary IJ. Reliability and validity of the UK Biobank cognitive tests. *PLOS ONE*. 2020;15(4):e0231627. doi:10.1371/journal.pone.0231627

7. Morys F, Dadar M, Dagher A. Association Between Midlife Obesity and Its Metabolic Consequences, Cerebrovascular Disease, and Cognitive Decline. *The Journal of Clinical Endocrinology & Metabolism*. 2021;106(10):e4260-e4274. doi:10.1210/clinem/dgab135

8. Maximov II, Alnæs D, Westlye LT. Towards an optimised processing pipeline for diffusion magnetic resonance imaging data: Effects of artefact corrections on diffusion metrics and their age associations in UK Biobank. *Human Brain Mapping*. 2019;40(14):4146-4162. doi:10.1002/hbm.24691

9. Veraart J, Fieremans E, Novikov DS. Diffusion MRI noise mapping using random matrix theory. *Magn Reson Med*. 2016;76(5):1582-1593. doi:10.1002/mrm.26059

10. Kellner E, Dhital B, Kiselev VG, Reisert M. Gibbs-ringing artifact removal based on local subvoxel-shifts. *Magnetic Resonance in Medicine*. 2016;76(5):1574-1581. doi:10.1002/mrm.26054

11. Andersson JLR, Skare S, Ashburner J. How to correct susceptibility distortions in spin-echo echo-planar images: application to diffusion tensor imaging. *NeuroImage*. 2003;20(2):870-888. doi:10.1016/S1053-8119(03)00336-7

12. Smith SM, Jenkinson M, Woolrich MW, Beckmann CF, Behrens TE, Johansen-Berg H. Advances in functional and structural MR image analysis and implementation as FSL. *NeuroImage*. 2004;23:S208-S219.

13. Andersson JLR, Graham MS, Drobnjak I, Zhang H, Filippini N, Bastiani M. Towards a comprehensive framework for movement and distortion correction of diffusion MR images: Within volume movement. *Neuroimage*. 2017;152:450-466. doi:10.1016/j.neuroimage.2017.02.085

14. Andersson JLR, Sotiropoulos SN. An integrated approach to correction for off-resonance effects and subject movement in diffusion MR imaging. *NeuroImage*. 2016;125:1063-1078. doi:10.1016/j.neuroimage.2015.10.019

15. Andersson JLR, Graham MS, Zsoldos E, Sotiropoulos SN. Incorporating outlier detection and replacement into a non-parametric framework for movement and distortion correction of diffusion MR images. *NeuroImage*. 2016;141:556-572. doi:10.1016/j.neuroimage.2016.06.058

16. N. J. Tustison, B. B. Avants, P. A. Cook, et al. N4ITK: Improved N3 Bias Correction. *IEEE Transactions on Medical Imaging*. 2010;29(6):1310-1320. doi:10.1109/TMI.2010.2046908

17. Smith SM, Jenkinson M, Johansen-Berg H, et al. Tract-based spatial statistics: voxelwise analysis of multi-subject diffusion data. *NeuroImage*. 2006;31(4):1487-1505. doi:10.1016/j.neuroimage.2006.02.024

18. Mori S, Oishi K, Jiang H, et al. Stereotaxic white matter atlas based on diffusion tensor imaging in an ICBM template. *NeuroImage*. 2008;40(2):570-582. doi:10.1016/j.neuroimage.2007.12.035
